# Nurses’ Professional Performance: The Development and Evaluation of a Formative Workplace-Based Self-Assessment Instrument

**DOI:** 10.1016/j.ijnsa.2026.100542

**Published:** 2026-05-14

**Authors:** Freda M.D. Vasse, Wim P. Krijnen, Marieke F. Van Der Schaaf, Evelyn J. Finnema

**Affiliations:** a Nursing Science and Education, Department of Health Sciences. University Medical Center Groningen, University of Groningen, P.O. Box 30.001, 9700 RB, Groningen, The Netherlands; b Founder, CEO, and owner of Frenetti®, Maastricht, The Netherlands; c University of Groningen, Faculty of Science and Engineering, The Netherlands; d Applied Statistics, Health Studies, Hanze University of Applied Sciences, The Netherlands; e University Medical Center Utrecht, Utrecht Center for Research and Development in Health Professions Education. Full Professor in Research and Development in Health Professions Education, The Netherlands; f Department of Health Science, Full Professor in Nursing Science and Education, University Medical Center Groningen, University of Groningen, Groningen, The Netherlands; g Member of the executive board, NHL Stenden University of Applied Sciences, Leeuwarden, The Netherlands

**Keywords:** Performance assessment, Nurse practice, Workplace measurement instrument, Nurses’ task expertise, Professional expertise, Professional development

## Abstract

**Background:**

Workplace-based performance assessment and feedback are essential for nurses’ development, helping to close the gap between their expertise and the requirements of their job roles and guiding the development of their expertise. Mismatches can lead to challenges, including lower-quality care, reduced job satisfaction, work stress, burnout, and early job turnover. In this study, a formative workplace-based performance assessment is developed to address this gap.

**Aim:**

To develop and evaluate a workplace-based, development-oriented performance assessment instrument concerning nurses’ professional tasks from beginner to expert levels.

**Design:**

Instrument construction followed an iterative design based on the Evidence-Centered Design (ECD) Model of Mislevy (2012). This approach addressed the ‘Eight Validity Challenges’ outlined by Mislevy (2016) related to the design, modeling, scoring, and scale development of nurses’ professional performance.

**Methods:**

The Formative Assessment for Nurses' Professional Performance (FAN) instrument was developed in the Netherlands using the Evidence-Centered Design Model and ‘Eight Validity Challenges’. Initial indicators were derived from participation, observation, literature searches, and analysis of nurses’ tasks. Several independent, experienced professionals reviewed the content and provided feedback. During development, multiple analyses were conducted to assess reliability and validity, including Exploratory Factor Analysis, reliability analysis, Item Content Validity, Pearson’s correlation, Generalized Partial Credit Model, Confirmatory Factor Analysis, independent-samples t-tests, ANOVA, and paired-samples t-tests.

**Results:**

The Formative Assessment for Nurses' Professional Performance instrument, developed using the Evidence-Centered Design Model, comprises three job domains, 21 subscales, and 93 items that span the broad scope of nurses’ work. It assesses nurses’ professional performance tasks from beginner to expert levels and identifies their development needs. The instrument was completed by 5,411 nurses across various roles and workplace settings in 22 hospitals and two rehabilitation centers in the Netherlands. It consistently demonstrated confirmatory factor loadings above .50, Cronbach’s alpha coefficients above .70, and discrimination coefficients above 1.0. Expected differences by educational level, job role, and workplace setting were tested statistically. All ‘Eight Validity Challenges’ for educational assessment development were integrated.

**Conclusion:**

This study provides strong evidence for the validity, reliability, and feasibility of the Formative Assessment for Nurses' Professional Performance among nurses across different educational levels, job roles, and workplace settings across a variety of hospitals and rehabilitation centers in the Netherlands. The workplace-based self-assessment measures nurses’ professional performance, identifies gaps, and supports professional development and workforce reform at both the team and organizational levels. It also provides direction for aligning organizational goals with nurses' goals.

**What is already known**

• The association of nurses’ educational level with patient outcomes.
• Nurse shortages with high turnover and burnout rates.

Alt-text: Unlabelled box dummy alt text

**What this paper adds**

• Clear differentiation of nurses’ professional tasks from beginner to expert levels.
• Validity evidence for measuring performance levels at both the vocational and bachelor’s levels.
• Instrument measurements to help meet future task demands and workforce reform.

Alt-text: Unlabelled box dummy alt text

## 1 Introduction

A strong nursing profession that involves reimagining and optimizing nursing is crucial to achieving Universal Health Coverage (Catton, 2025). Factors such as population aging, chronic diseases, technological advances, and a shortage of skilled nurses—along with high rates of intentional turnover and burnout—underscore the urgent need for substantial workforce reform (Aiken et al., 2016; Griffiths et al., 2023; Hussain et al., 2012; Michaeli et al., 2024; Wang et al., 2024; World Health Organization, 2025). Nursing shortages result in consequences, including increased medication errors, infection risks, and patient falls (Catton, 2025).

Addressing these challenges requires a comprehensive understanding of the complexity and diversity of tasks performed by nurses as well as expertise development (Humburg & Van der Velden, 2017).

The development of nurses’ expertise can be described as an adaptation to task demands in the work environment. This adaptation involves restructuring, reorganizing, and refining how they represent knowledge and procedures to apply them efficiently in daily work (Feltovich et al., 2018), where expertise is defined as the outcome of a prolonged period of learning (Ericsson, 2018). To increase nurses’ sense of expertise, achievement, and fulfillment, organizations need to help nurses create meaning and purpose in their professional development and in pursuing specialized expertise. These strategies increase job satisfaction, job embeddedness, and engagement, reduce intent to leave or turnover, and reduce nurses’ burnout (Wang et al., 2024; Wei et al., 2023), and foster a workplace that recognizes, values, and uses the strengths of all employees (Smith, 2017; Wei et al., 2023).

Fundamentals include clear job and task descriptions, adequate differentiation of nurses’ tasks in workplace settings, and long-term career development plans for professional growth (Poitras et al., 2016; Pursio et al., 2021), aligned with the demands and expectations of clients and colleagues (Eraut, 2008; Filliettaz, 2014). Sufficient nursing expertise is a prerequisite for favorable patient outcomes (Aiken et al., 2002; Aiken et al., 2016; Ball et al., 2018; Needleman et al., 2011; Twigg et al., 2019). Task complexity lies in the interplay among the physical, emotional, cognitive, and organizational aspects of nurses’ work, as well as the degree of professional autonomy (Jackson et al., 2021; Pursio et al., 2021). Job performance is conceptualized as both contextual and adaptive performance within tasks (Krijgsheld et al., 2022).

The current state of nursing workforce development calls for a reliable and valid performance assessment instrument that covers the full range of nurses’ tasks across various workplace settings, from beginner to expert levels. The absence of such an instrument can lead to mismatches between nurses’ skills, job requirements, and opportunities for growth (Smith, 2017).

Currently used generic instruments to assess nurses’ competence include the Nurse Competence Scale (NCS) (Meretoja et al., 2004), the Nurse Professional Competence Scale (NPCS; a shorter version of the NCS) (Nilsson et al., 2018), the Competence Inventory for Registered Nurse (CIRN) (Liu et al., 2009), the European Questionnaire Tool (EQT1 and EQT2) (Cowan et al., 2008), and the Holistic Nursing Competence Scale (HNCS) (Takase & Teraoka, 2011). These generic competency assessments share subscales across the physical, emotional, cognitive, and organizational domains of nurses’ work and professional development (Jackson et al., 2021). In these instruments, measurement is per item, often featuring a competence or performance description as a statement, with scoring using a Likert-style response such as: ‘never’ to ‘always’, from a ‘very low’ to a ‘very high’ degree, or ‘not at all’ to ‘to a very great extent’ (e.g., Charette et al., 2020; Flinkman et al., 2017). A higher score indicates greater use of the competence described in the item. An option is provided for respondents who are unable to answer. These Likert-style answer categories differ from concrete constituent performance task descriptions at the behavior level, which are used to measure expertise from beginner to expert.

Competence assessment tools are primarily self-report measures that focus on competence rather than actual professional performance in real-world settings. Generally, there is limited emphasis on task authenticity, contextual variability, or developmental progress from beginner to expert. Therefore, there is a clear need for an assessment instrument that provides authentic, formative, and developmental evaluation of nursing performance across different roles and contexts, and that differentiates among various types and levels of nurses (World Health Organization, 2025). This highlights the need to develop a new instrument for formative assessment of nurses’ professional performance.

In professional contexts, competence and performance are closely related yet conceptually distinct. Competence is commonly defined as what an individual knows and can do under ideal circumstances (Messick, 1994; While, 1994). In nursing, competence entails the integrated use of knowledge structures, complex cognitive and higher-order skills, interpersonal and social skills, attitudes, values, and professional judgment to deliver safe and effective care across diverse situations (Cowan et al., 2008; Mrayyan et al., 2023). By contrast, performance refers to what professionals actually do in real practice under authentic conditions. It is the observable manifestation of underlying, latent competence. Professional performance is therefore context-sensitive and dynamic, shaped by workplace demands, patient complexity, the organization’s arrangements for professional work, and the individual’s level of expertise (Eraut, 1994). While competencies may be transferable across roles, tasks, and situations, performance is expressed in concrete actions within specific professional contexts. Following Eraut’s conceptualization, performance in this study is understood as the enactment of acquired competencies in everyday professional practice, within established frameworks for nurse performance, and aligned with the International Council of Nurses' (2025) contemporary redefinition of the nursing profession.

In instrument development, the distinction between competence and performance has implications for assessment methodology (Chomsky, 2006). The Evidence-Centered Design (ECD) Model is a framework for systematically designing assessments by explicitly linking constructs, evidence, and tasks (Mislevy, 2012). Its theoretical foundation lies in construct validity and argument-based assessment (Messick, 1994). ECD comprises four core components: the Domain Model (specifies the constructs to be assessed, meaning what is to be measured), the Task Model (which involves selecting of essential tasks and their associated contexts in which nurses are required to demonstrate evidence of their expertise and performance), the Presentation Model (which covers the final assessment instrument, including how results are displayed and visualized), and the Evidence Model (analyzes observed performances, meaning observable behaviors, in terms of validity and reliability, as well as their intended interpretation and use). ECD is highly relevant to performance assessment because it supports the design of authentic, real-world tasks and ensures valid inferences about professional performance. Next, the ‘Eight Validity Challenges’ provide guidance in assessment development to harness nurses’ capabilities in the design process. These challenges are: 1) What do we want to assess? 2) What kinds of performances do we need to observe, and in what kinds of situations? 3) How should we think about constructs? 4) How do we assess higher-order skills? 5) What is the role of measurement models? 6) How do we “score” complex, interactive performance at scale? 7) How do we assess interactional skills? 8) How do we take advantage of complex performance tasks? (Mislevy, 2016).

This study aims to develop and evaluate a workplace-based, development-oriented performance assessment instrument concerning nurses’ professional tasks across all levels from beginner to expert. It then details how the Formative Assessment for Nurses’ Professional Performance was developed and evaluated using the Evidence-Centered Design Model (Mislevy, 2012) and the ‘Eight Validity Challenges’ (Mislevy, 2016).

## 2 Method

### 2.1 Design

The Formative Assessment for Nurses' Professional Performance (FAN) was developed in the Netherlands and underwent multiple iterative design stages, guided by the ECD (Mislevy et al., 2012). The instrument was evaluated along the ‘Eight Validity Challenges’ related to the design, modeling, scoring, and scale development of nurses’ professional performance, as outlined by Mislevy (2016).

The first author developed the instrument based on extensive, relevant experience in various roles and work environments, including clinical and outpatient settings. This background encompasses general and specialized bedside nursing, nurse education, healthcare innovation, and educational science and design. By combining this nursing and healthcare experience with reflexivity, 'matrix thinking,’ and visualization, the author integrated additional literature searches and investigations to create items and performance levels aligned with the study's theoretical concepts and framework.

The developed items and answer categories are evaluated by a group of experienced nurses, thereby establishing the assessment instrument’s content validity.

### 2.2 Stage 1) Domain Model – domain identification of topics in nursing practice

The first stage began by creating an initial list of topics relevant to nurses’ work, characterizing nurses’ job domain and expertise across various work settings (Eraut, 2008). The Canadian Medical Education Directives for Specialists (CanMEDS) framework (Frank & Danoff, 2007) was selected as a starting point because it is central to Dutch professional nursing profiles (Schuurmans, 2012) and is integrated into Dutch bachelor’s nursing programs (Bouwes et al., 2023). The seven CanMEDS domains were originally developed for medical professionals (Frank & Danoff, 2007). The professional nursing profiles framework (2012) comprises seven similar domains tailored for bachelor’s nurses, namely: 1) Nurse Expert; 2) Communicator; 3) Collaboration Partner; 4) Reflective ‘Evidence-Based Practice’ (EBP) Professional; 5) Health Promoter; 6) Organizer/Leader; 7) Quality Promoter. The national Bachelor of Nursing education profile for 2020 and 2030 in the Netherlands, aligned with the seven CanMEDS domains, outlines nurses’ competencies, elaborated into 24 core concepts (Bouwes et al., 2023). Only the previously mentioned CanMEDS domain names served as an overarching framework at the start of the first stage of instrument development. A universal approach centered on the care receiver from a nurse professional's perspective was adopted.

Next, topics for potential FAN items were identified through participation, observation, literature searches, investigations, and analysis of nurses’ tasks. These topics were then assigned to the most appropriate CanMEDS domain description for nurses.

For each CanMEDS domain, the relevance of topics was assessed using a convenience sample of four experienced nursing professionals working in different hospitals across the Netherlands. They were asked to verify the topic list, which served as the foundation for the FAN instrument. Then, the topics were operationalized into items.

#### 2.2.1 Participants of the Item Content Validity survey and procedure

Additionally, an Item Content Validity investigation was conducted by distributing a survey to a convenience sample of participants with relevant backgrounds to evaluate the relevance of all items. Inclusion criteria were: 1) working in a nursing context with a nursing background, or 2) working in nurse education. Participants rated each item's content as ‘essential,’ ‘useful, but not essential,’ or ‘not essential’ for vocational- and bachelor-educated nurses. Consequently, each item received one rating for the vocational-educated nurse and another for the bachelor-educated nurse. In addition to examining frequencies, a paired-samples t-test, in which each rater forms a pair, was performed to analyze differences in item relevance between vocational- and bachelor-educated nurses.

### 2.3 Stage 2) Task Model – development of task-level descriptions

Stage two comprised descriptions of task levels for the 1) beginner, 2) competent, 3) proficient, and 4) expert (Benner, 1982; Dreyfus, 2004).

The beginner level indicates basic competence, where nurses may need assistance to work independently. The competent level reflects adequate performance in common standard situations. The proficient level involves handling complex situations. The expert level involves metacognitive abilities, synthesis, and advanced problem-solving skills, including nurse leadership (Benner, 1982; Dreyfus, 2004; Eraut, 2008).

The performance level descriptions are created for each item through four steps:

**Step 1)** Investigation of the content of nurses’ work to create constituent ‘Whole Tasks’ for each item, arranged in a sequence from performance level one to four, following the first component of the ‘Four-Component Instructional Design’ (4C/ID) Model (Van Merriënboer & Kirschner, 2018). ‘Whole Tasks’ refer to nurses’ work from a holistic design perspective, as defined in the ‘Four-Component Instructional Design’ Model (Francom & Gardner, 2014; Van Merriënboer & Kirschner, 2018; Francom, 2018), carried out across different contexts. Task levels 1 to 4 are used to structure the job content of professional tasks from standard (non-complex, routine, predictable) to complex (non-routine, unpredictable) (Van Merriënboer & Kirschner, 2018). The key job content served as input to create ‘Whole Task’ descriptions of constituent knowledge, skills, and attitudes, ranging from beginner to expert. This was done through task decomposition, starting at the proficient level, advancing to the competent level, and then to the beginner level. The expert level incorporates additional leadership abilities from a metacognitive perspective, including synthesis and advanced problem-solving skills (see Table 1, step 1).

**Table 1:** Four steps with content standards criteria for creating task levels descriptions from beginner to expert.

Steps	Task levels aligned with content standards criteria from beginner to expert			
	1) Beginner criteria	2) Competent criteria	3) Proficient criteria	4) Expert criteria
**Step 1)** Job content investigation per topic/item to create ‘Whole Tasks’ level 1 to 4 sequencing according to 4C/ID Model 1 (Van Merriënboer & Kirschner, 2018)	Task level 1: most simple form of the job content and task	Task level 2: intermediate form of the job content and task	Task level 3: more complex form of the job content and task	Task level 4: most complex form of job content and task
**Step 2)***Revised* Taxonomy of Bloom with the list of ‘action verbs’ per cognitive category/dimension (Kratwohl & Anderson, 2001)	Lower-order thinking skills: I) Remembering, II) Understanding, facts (reproduction of).	Lower-order thinking skills: III) Application of facts (i.e., knowledge) and concepts.	Higher-order thinking skills: IV) Analyze and V) Evaluate procedures.	Higher-order thinking skills: To VI) Create and metacognition.
**Step 3)** Workplace level of task complexity (Van Merriënboer & Kirschner, 2018)	Standard situation	Standard situation	Complex situation	Complex situation
**Step 4)** Social interaction inter/intraprofessional (Eraut, 2008)	Individual: Identifying and asking for help or support	Individual, group: Identifying and asking for help or support	Individual, group/ team/multidisciplinair: Identifying and offering help or support	Team, group/multidisciplinary/organization broad/ professional leadership at the team level: Identifying and offering help or support

1 ‘Four-Component Instructional Design’ Model (4C/ID) of Van Merriënboer & Kirschner (2018).

For each item, relevant job content information was collected from a ‘Whole Task’ perspective (see Table 1, step 1) and used as input for the task descriptions for levels 1 to 4. We took into account as much as possible a variety of word usage in the instrument to avoid language monotony.

**Step 2)** For each task description used as an answer category from beginner to expert, ‘action verbs’ from the *revised Bloom's* Taxonomy were integrated (Krathwohl, 2002; Anderson & Krathwohl, 2001). Examples of ‘action verbs’ include ‘to classify,’ ‘to apply,’ and ‘to analyze.’ An ‘action verb’ describes what nurses can do and must indicate observable, attainable, and measurable behaviors (Van Merriënboer & Kirschner, 2018) (see Table 1, step 2). ‘Action verbs’ can belong to lower or higher (cognitive) levels. That is, I. Remembering, II. Understanding, and III. Applying involves categories with ‘action verbs’ of lower order, while IV. Analyzing, V. Evaluation, and VI. Creating involves categories with ‘action verbs’ of higher-order thinking skills (Anderson & Krathwohl, 2001). These different categories of ‘action verbs’ were accordingly integrated into the four task descriptions, ranging from beginner to expert levels.
**Step 3)** Next, where applicable in the task description, indicate whether the professional task occurs in a standard or complex situation according to the ‘Four-Component Instructional Design’ (4C/ID) Model (Van Merriënboer & Kirschner, 2018). Workplace complexity can be defined as the degree to which nurses encounter routine (non-complex) or unpredictable non-routine situations (complex), or as the level of task complexity (Van Merriënboer & Kirschner, 2018) (see Table 1, step 3).

When applicable to the topic, ‘standard situation’ was used in the task descriptions at the beginner and competent levels, and ‘complex situation’ at the proficient and expert levels.

**Step 4)** The social interaction, knowledge transfer, and discourse about work between nurses and others involved in nurses’ work were integrated into the task descriptions, following Eraut (2008), because nurses’ work relies on teamwork and mutual support is essential (see Table 1, step 4). Progress from beginner to expert can be achieved through scaffolding, moving from full support and partial support to no support (Van Merriënboer & Kirschner, 2018), and through the ‘Zone of Proximal Development in the workplace’ (Filliettaz, 2014; Vygotsky, 1978). This area, between what a nurse can already do independently and what is not yet possible without support, can be developed with guidance, such as from a colleague with more expertise or a supervisor.

We examined how the four steps are used in task descriptions at the beginner, competent, proficient, and expert levels. This was to determine if they meet the criteria for a cumulative scale, as shown in Table 1.

### 2.4 Stage 3) Presentation Model – development of job domains and subscales

Stage three involved grouping items with the four task-level descriptions into content-based subscales. Exploratory Factor Analysis (EFA), Cronbach’s alpha estimates, and Pearson’s correlations were used to assess the quality of the initial subscales and, when necessary, refine them (Goretzko et al., 2024). Occasionally, items that fit better according to the EFA and Pearson’s correlations were moved to a different subscale and the related CanMEDS domain. A content-based decision was made when an item loaded on more than one factor, given the requirement of at least three items as mentioned earlier.

Items with factor loadings >.50 contributing to a Cronbach’s alpha for internal consistency >.70 (Nunnally, 1967; Cant et al., 2013), and Pearson’s correlations in a positive direction with *r* > .50 (p < 0.01), which are classified as ‘moderate’ correlations, were selected as subscales of the FAN. However, when Pearson’s correlation was *r* < .50, and the factor loading was >.50, the subscale was retained. A name was assigned to each subscale, reflecting the content of the items.

Next, subscales were grouped into three job domains based on the content of each subscale and the purpose of each domain, that is: 1) direct care, corresponding to the central CanMEDS domain of Nurse Expert; 2) socio-cognitive and socio-constructive practices, including Linguistic, Cultural and Substantive (LCS) patterns, demands and expectations, corresponding to the CanMEDS domains of Communicator and Collaboration Partner; and 3) indirect care, corresponding to the CanMEDS domains of Reflective ‘EBP’ Professional, Health Promoter, Organizer/Leader, and Quality Promoter. Cronbach’s alpha and Pearson’s correlation were computed across the three job domains.

### 2.5 Stage 4) Evidence Model – Construct validation

#### 2.5.1 Participants of the FAN and procedure

The data for this study were collected using convenience sampling from 2016 to 2023 among registered nurses working in 22 hospitals and two rehabilitation centers in the Netherlands. They worked in various departments, such as acute care, surgical and non-surgical, clinical, and non-clinical, and were invited to participate in professional development projects or programs using the performance assessment instrument.

The invitation to participate was issued by the head or team leader of the patient department (the nurses’ workplace), the Human Resources department, or the Academy department. It was shared collectively and sent in writing through the internal system, depending on the structure, processes, and size of the hospital or rehabilitation center.

Participants were asked to rate their performance on each item in the FAN, which reflects their level of expertise and, if they choose, their professional development goal. Items unrelated to the nurse’s job role or workplace setting were marked as ‘not applicable’. Nurses completed the performance assessment during a shift at their workplace, in a computer classroom, or at home. The software application allowed them to revisit items to make adjustments, pause, and resume later or on another day. Participants completed the assessment with their work and actual performance in mind.

#### 2.5.2 Ethical considerations

Participants who accessed the software application to complete the assessment were required to give informed consent regarding the privacy statement and terms of use, including for scientific purposes. Later, the data were used formatively in a meeting with their team leader for professional and career development and were kept confidential. Non-participation did not put nurses in our population at risk. The data were pseudonymized.

#### 2.5.3 Data analysis

The Generalized Partial Credit Model was used as an item response model, allowing cumulative characteristics to be evaluated across a set of responses, with responses ranked from “easiest” (beginner) to “most difficult” (expert) (discrimination coefficients > 1.0) (Bürkner et al., 2019). This is combined with EFA factor loadings (threshold of EFA factor loadings >.50, indicating strong when sample size is large) and Cronbach's alpha (threshold of coefficients >.90: Excellent; 0.8 ≤ α < 0.9: Good; 0.7 ≤ α < 0.8: Acceptable; 0.6 ≤ α < 0.7: Questionable; 0.5 ≤ α < 0.6: Poor; α < 0.5: Unacceptable) (Nunnally, 1967; Cant et al., 2013; Sathyanarayana & Mohanasundaram, 2024; Watkins, 2018), using the statistical programming language R (R Core Team, 2021).

A second-order Confirmatory Factor Analysis (CFA) is used to evaluate the hypothesized three-factor structure consisting of the three job domains: JD1 Nursing & Patient Care, JD2 Collaboration & Communication, and JD3 Managing & Improving, along with an overarching one-factor structure representing nurses’ work. First, the normality of the subscales was tested using the Kaiser-Meyer-Olkin (KMO) measure (desired values ≥.70) (Watkins, 2018), following the estimation methods in structural equation modeling, such as weighted least squares and maximum likelihood (Rosseel, 2012). Next, CFA factor loadings were examined, with thresholds set at either standardized factor loadings greater than >.50 or > .40 to be considered reliable and meaningful for interpretation; in relatively large samples, with 200 or more cases, a construct explains at least > .25 (Cheung et al., 2023). CFA correlations were also evaluated, with threshold values of r ≥ 0.5 indicating a large correlation; between ≤ 0.3 and < 0.5 denoting medium; between ≤ 0.1 and < 0.3 indicating small; and < 0.1 representing very small or no correlation (Cohen, 1988). The four commonly used fit indices were evaluated: the Comparative Fit Index (values above 0.90 indicate good fit), the Tucker-Lewis Index (values above 0.90 indicate good fit), both non-normed fit indices; the Root Mean Square Error of Approximation (values less than 0.05 considered indicative of good fit, while values up to 0.08 are considered reasonable in some contexts); and the Standardized Root Mean Square Residual (values less than 0.08 as indicative of good fit). These indices quantify the goodness of fit and the deviation from a perfect model fit given the data (Goretzko et al., 2024; Sathyanarayana & Mohanasundaram, 2024).

Furthermore, as part of the expected construct evidence in ECD stage four, independent-samples t-tests are conducted to compare the mean differences between vocational- and bachelor-educated nurses. Next, one-way ANOVA tests and the Tamhane method (Tamhane, 1979) with p-values are used to assess differences in the expected means across job roles and workplace settings. Paired-samples t-tests are used to examine mean differences in nurses' performance levels and their professional development goals. All these tests are conducted using SPSS 28. Cohen’s d effect size interpretation is: > 0.2, small effect; > 0.5, medium effect; > 0.8, large effect (Cohen, 1988).

Finally, the ‘Eight Validity Challenges’ are evaluated.

## 3 Results

### 3.1 Stage 1) Domain Model – domain identification of topics in nursing practice

#### 3.1.1 Collection of topics and expert review process

In stage one, topics were collected based on nurses' relevant proven expertise. The initial 77 items chosen in 2013-2014 were organized within the CanMEDS seven-domain framework. The expert review process kept most of these items unchanged. In 2015-2016, 19 additional items were added to cover the scope of nurses’ work. All 96 items were verified through several rounds of evaluation by experienced professionals in the Netherlands. Two items were removed due to overlap, and one was omitted because it was not directly related to the workplace. This process resulted in a FAN instrument with 93 items.

#### 3.1.2 Item Content Validity Survey

Data collection for the Item Content Validity took place in 2016. The survey was conducted with a convenience sample of 162 professionals. A broad group of 103 (64%) professionals participated, of whom 94 (91%) worked in various hospitals and 9 (9%) in other healthcare organizations or educational institutes. Nearly all participants (102, 99%) had a nursing background. Of these, 60 (58%) worked in direct care, while 43 (42%) were involved in indirect care, such as management or education.The results of the Item Content Validity survey confirmed the relevance of all items in the FAN instrument for both vocational- and bachelor-educated nurses. Paired-samples t-tests showed that relevance ratings were significantly higher among nurses with a bachelor’s degree compared to vocational-educated nurses. According to Cohen’s d, the effect size was small (Cohen, 1988) (see Table 2).

**Table 2:** Mean (M) and Standard Deviation (SD) of the relevance ratings, along with paired-samples t-test and Cohen’s d, for the items across the three job domains, JD1, JD, JD3, with n=103 participants (raters) for the vocational and bachelor educational levels of nurses. Scoring range: 1, ‘Not essential’; 2, ‘Useful, but not essential’; and 3, ‘Essential’.

	*Vocational level*		*Bachelor level*		*Paired-samples t-test*		
	M	SD	M	SD	*T*	P*	*Cohen’s d*
JD1: Nursing & Patient Care	2.9	.10	3.0	.09	-4.10	<.001	.10
JD2: Collaboration & Communication	2.9	.14	3.0	.08	-6.35	<.001	.15
JD3: Managing & Improving	2.7	.21	3.0	.07	-11.93	<.001	.20

⁎ P two-sided significance p <.001

### 3.2 Stage 2) Task Model – the creation of task-level descriptions from beginner to expert

In the second stage, the four answer categories for each item from the list in stage one were developed, guided by the four steps shown in Table 1.

**Step 1)** The task content and readability were reviewed by four experienced professionals from four Dutch hospitals, each with 10 to 30 years of experience. Among them were two senior nurses, one specialized nurse with a background in educational sciences and teaching experience, and one specialized senior nurse with research experience. They recommended minor linguistic changes to about 20% of the task-level descriptions. For example, they suggested changing the wording from ‘good’ to ‘sufficient’ for a specific answer category. They also proposed revising terminology in some answer categories, such as using ‘multidisciplinarity’ instead of ‘other disciplines’.
**Step 2)** Table 1 in the supplementary Appendix presents the subtotal and total counts of ‘action verbs’ for each category from the *revised* Bloom’s taxonomy (Anderson & Krathwohl, 2001) in the professional task-level descriptions. ‘Action verbs’ are spread across the categories ‘Remembering,’ ‘Applying,’ ‘Analyzing,’ ‘Evaluation,’ and ‘Creating,’ and are well represented across the answer categories. Within ‘Understanding,’ only one ‘action verb’ was used. The table shows that at the beginner and competent levels, ‘action verbs’ are mainly used in the lower-order thinking skills category, while at the proficient and expert levels, ‘action verbs’ are more often used in the higher-order thinking skills category. As expected, ‘action verbs’ vary across task levels one to four according to the *revised* Bloom’s taxonomy.
**Step 3)** The level of task complexity in the work environment was integrated with the professional task descriptions from beginner to expert (see Table 1, step 3). This meant that, where appropriate, the description of ‘standard situation’ appeared in the task description 12 times at the beginner level and 13 times at the competent level. References to ‘complex situation’ were generally included in items of the task description at the proficient level.
**Step 4)** In this step, characteristics related to ‘social interaction’, such as ‘giving support’, ‘discussing’, or ‘consultation’, mainly appeared in the expert-level task descriptions, totaling 180 times. This was followed by the proficient level (81 times), the beginner level (63 times), and the competent level (46 times). Conversely, the phrase to ask for ‘help’ appeared 25 times at the beginner level and five times at the competent level.

Knowledge transfer, discourse about work, discussions of expertise, and scaffolding, such as decreasing guidance, are integrated across levels from competent to expert in initiating, supporting, consulting, and discussing professional tasks with colleagues.

Altogether, steps 1, 2, 3, and 4 resulted in final professional task descriptions for each item, ranging from beginner to expert levels.

### 3.3 Stage 3) Presentation Model – Construction of the Performance Assessment Instrument

This stage led to the construction of the FAN instrument. Over the years, multiple analyses have been conducted to identify latent variables that represent nurse performance. These evaluations, including Exploratory Factor Analysis (EFA) to examine a set of items related to a construct, empirical findings from previous studies on sub-data sets, theoretical and content-based considerations, explained variances, Cronbach’s alpha calculations, and Pearson’s correlations on sub-data sets, led to the development of 21 subscales (i.e., rubrics). The item-to-factor ratio is as follows: five subscales contain three or five items, seven contain four items, three contain six items, and one contains seven items. These initial subscales reflect a range of themes in nurses’ work, from ‘simple’ to 'complex,’ capturing real-life professional ‘Whole Tasks.’ Using Cronbach’s alpha and Pearson’s correlation, the 21 subscales were further grouped into three job domains, categorized as nurses’ work related to: 1) direct care; 2) linguistic, cultural, and substance social practices; and 3) indirect care, collectively representing the ‘Whole Task’ of nurses’ work in the FAN instrument.

Job domain 1, ‘Nursing & Patient Care,’ includes nine subscales with 41 items. It relates to direct care and aligns with the initial list within the CanMEDS domain of Nurse Expert, as established in stage one.

Job domain 2, ‘Collaboration & Communication,’ includes six subscales with 24 items and covers interactions with patients, next-of-kin/informal caregivers, colleagues, management, administration, and other healthcare professionals. It reflects social practice from socio-cognitive and socio-constructive perspectives, including linguistic, cultural, and substantive patterns. It aligns with the two similar CanMEDS domains, 'Communicator' and 'Collaboration Partner.'

Job domain 3, ‘Managing & Improving,’ includes six subscales with 28 items and relates to indirect care. It can be viewed as the overall organization and enhancement of care. Indirect care aligns with the four CanMEDS domains: the Reflective ‘EBP’ professional, the Health Promoter, the Organizer/Leader, and the Quality Promoter.

Due to the continued use of the ‘Whole Task’ approach within the ‘Four-Component Instructional Design’ (4C/ID) Model (Van Merriënboer & Kirschner, 2018), the seven CanMEDS domains are assigned to the three job domains of the FAN.

The Formative Assessment for Nurses' Professional Performance (FAN) instrument comprises three job domains, 21 subscales, and 93 items, as shown in Table 3.

**Tabel 3:** Construct formation with the description of the three job domains, the 21 subscales and 93 items within the Formative Assessment for Nurses' Professional Performance instrument (FAN).

Job domain 1: Nursing & Patient Care	
Subscales (k=9)^2^	Items (k=41)
Nursing process	Principles of the nursing process; Admission, care needs and intervention; Drawing up nursing care plans; Nursing diagnoses; Outcome of nursing care plan; Hand-off and reporting procedures (care documentation). (k=6)
Technical skills and risk-awareness	Reserved and high-risk procedures; Technical nursing skills and procedures; Medical devices and support equipment. (k=3)
Safety and prevention	Hygiene regulations for prevention; Addressing my own safety and that of others; Dealing with hospital waste; Working hygienically when providing ADL (Activity of Daily Life) care. (k=4)
Medical knowledge	Anatomy – physiology; Somatic diseases, disorders and impairments; Pharmacology – Pharmacotherapy; Psychiatric diseases, disorders and impairments (k=4)
Identifying urgency of care	Recognizing symptoms; Identifying complications; Clinical reasoning; Unexpected and unstable situations (crisis intervention); Supervision and support with psychosomatics (k=5)
Critical nursing procedures	Decisions and nursing procedures; Recording and accountability of nursing actions; Evaluation and reflection with patients and/or next-of-kin. (k=3)
Patient or client focus	Hospitality; Relationship with the patient and/or next-of-kin; Patient instruction and information; Intercultural interaction. (k=4)
Service in healthcare	Coordinating care with patients; Self-management support and interventions; Self-management support with the aid of informal carers; Palliative care (prevention and alleviation of suffering); Encouraging relatives and informal carers to help; Eliciting help from relatives and informal carers. (k=6)
ICT skills and eHealth	ICT (Information Communication Technology) skills and applications; Use of electronic health records (EHRs); Use of ICT (Information Communication Technology) for remote care (telehealth); Organization of remote patient care (telehealth); Patients' self-monitoring and self-diagnosis; Use of computer programs. (k=6).

Job domain 2: Collaboration & Communication	
Subscales (k=6)	Items (k=24)
Information processing	Acquiring information; Exchanging information; Listening skills. (k=3)
Communication techniques and skills	Communication skills; Structuring conversations; Reaching the goals and/or consensus in conversations. (k=3)
Patient-safe communication	Giving feedback; Receiving feedback; Calling colleagues to account; Opening up (near miss) incidents for discussion. (k=4)
Collaboration (inter-/multidisciplinary)	Teamwork; Peer consultation meetings; Inter-/multidisciplinary meetings; Multidisciplinary collaboration; Leadership tasks; Dealing with moral dilemmas; Team meetings. (k=7)
Compassion and empathy	Compassion and empathy awareness; Anticipating and responding to needs; Dealing with suffering and loss. (k=3)
Organizational process of care	Knowledge of one's own organization; Knowledge of other organizations involved in the care; Task differentiation/ reallocation; Collaboration in the organizational process of care. (k=4)

Job domain 3: Managing & Improving	
Subscales (k=6)	Items (k=28)
Coordination of care	Estimating the necessary care; Time management and planning; Continuity of care; Cost-awareness, avoiding waste; Care coordination. (k=5)
Patient safety culture	Patient safety and Patient Safety Management System; Dealing with conflicts with patients and /or next-of-kin; Dealing with conflicts with colleagues and other staff; Preventing mistakes and near misses. (k=4)
Quality of care	Guidelines, professional standards and protocols; Clinically uncertain situations and Evidence-Based Practice (EBP) nursing; Quality systems; Quality of care; Measuring quality. (k=5)
Social action	Epidemiology; Risk prevention and health education; Identifying ethical and moral dilemmas; Developments in society relevant to healthcare. (k=4)
Knowledge and science	Evidence-Based Practice; Looking up information on Evidence and Evidence-Based protocols; Current scientific developments; Review of nursing research and EBP articles; Multidisciplinary scientific medical research. (k=5)
Professionalism	Reflecting on your own actions and knowledge; Increasing expertise; Guiding and supporting new colleagues and students; Professional code in nursing; Legislation and regulations. (k=5)

2 k equals the number of subscales or items.

### 3.4 Stage 4) Evidence Model - Construct validation

#### 3.4.1 Characteristics of FAN Participants

Data collection with the FAN took place from 2016 to 2023 and involved 6,147 nurses, with a non-response rate of 337 (5.5%). Non-response reasons included sick leave, lack of time, or lack of motivation. Additionally, nurses with item non-response were excluded, leading to the removal of 399 (6.5%) nurses who started the FAN but did not finish it. In total, 5,411 (88%) nurses completed the assessment. Nurses were asked to identify each item's performance level and professional development goal. Data were collected from two University Medical Centers (2.3%), ten Top Clinical Teaching Hospitals (72.7%), ten General Hospitals (23%), and two Rehabilitation Centers (2%) in the Netherlands. The characteristics of the nurse participants (n=5,411) used for the construct validity of the FAN in this study are shown in Table 4 and include age groups, job roles, highest level of nursing education, years of workplace experience, and work setting.

**Table 4:** Characteristics of the nurse participants (n=5,411) for the construct validity of the Formative Assessment for Nurses' Professional Performance (FAN) instrument.

Variable		n	%
Age categories	< 30 years	1567	29
	30 – 40 years	1076	19.9
	40 – 50 years	969	17.9
	> = 50 years	1264	23.4
	missing	535	9.9
Job roles	General nurse	2946	54.5
	Specialized nurse	1764	32.6
	Senior nurse	403	7.4
	Nurse coordinator	208	3.8
	Nurse consultant	57	1.1
	Nurse practitioner	32	0.6
	Missing	1	0.0
Highest level of nurse education	Vocational level	3286	60.7
	Associated degree level	56	1.0
	Bachelor level	2051	37.9
	Higher than bachelor level	15	0.3
	Missing	3	0.1
Years of experience in the workplace setting	< 5	2425	44.8
	5 – 15	1439	26.6
	15 – 25	915	16.9
	> = 25	622	11.5
	Missing	10	0.2
Work hours per week by employee contract	< = 16 per week	346	6.4
	17 – 24	1952	36.1
	25 - 32	2346	43.4
	> = 33	735	13.6
	Not applicable	18	0.3
	Missing	14	0.3
Workplace setting	Acute care (Intensive care, Emergency room, Cardiac care unit)	485	9.0
	Surgical Care (including Recovery)	1195	22.1
	Non-surgical medical care	1716	31.7
	Mother - child - woman care	802	14.8
	Acute admission department care	110	2.0
	Dialysis care	171	3.2
	Rehabilitation care	118	2.2
	Psychiatric care	73	1.3
	Geriatric care	109	2.0
	Day care/treatment care	99	1.8
	Short stay care	45	0.8
	Out patient department care	187	3.5
	Flex pool (Flex pool means nurses without a fixed workplace)	187	3.5
	Other	114	2.1

#### 3.4.2 General Partial Credit Model

**Fig. 1:**
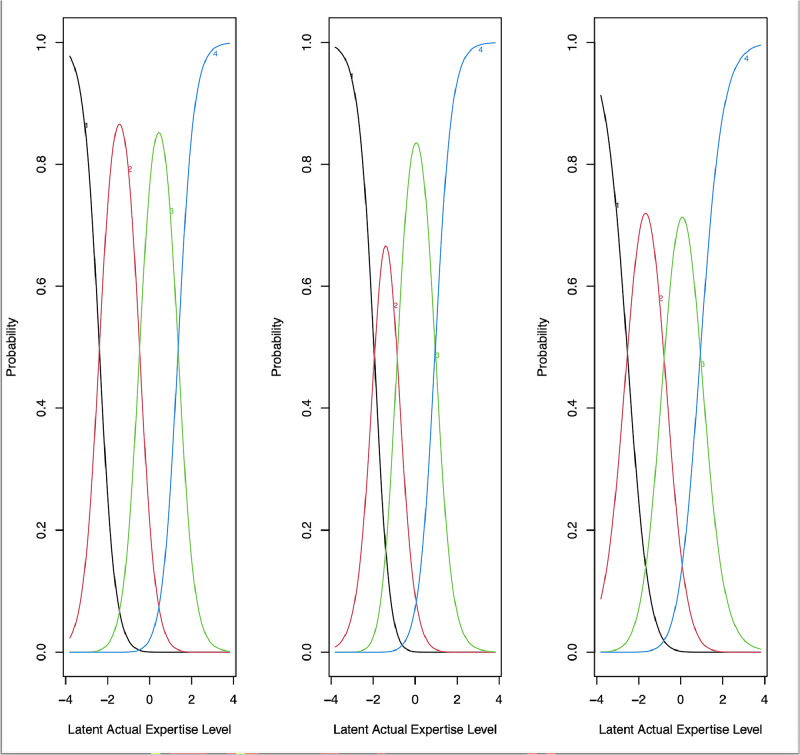
The characteristic curves of the subscale 'Critical nursing procedures’ show the vertical probability of an affirmative response for each item category, given the level of the nurse’s latent expertise scale (horizontal axis). The items, from left to right, are: 1) Decisions and nursing procedures; 2) Recording and accountability of nursing procedures; 3) Evaluation and reflection with patients and/or next of kin. The black curves represent the beginner level, the red curves the competent level, the green curves the proficient level, and the blue curves the expert level.

Cronbach’s alpha ranged from 0.8 to 0.9 for 14 subscales, indicating good reliability, and from 0.7 to 0.8 for 7 subscales, indicating acceptable internal consistency. Factor loadings for all 93 items exceeded .50, demonstrating strong loadings. Testing all 93 assessment items with the Generalized Partial Credit Model showed that the categories increase monotonically with the latent performance level trait. All category threshold parameters increase with the cumulative scale, covering a broad range of latent expertise. All discrimination parameters for the items were above 1.0 (1.03-4.85), except for one: the item ‘Organization of remote patient care (telehealth)’ at 0.89, as shown in the supplementary Appendix, Table 2. Next, the probability of responding in each category was estimated. For example, the curves in Fig. 1 display the item probability curves for each category within the subscale ‘Critical nursing procedures,’ showing the probability of an affirmative response given the nurse's latent scale level on the horizontal axis. For this same subscale, Table 5 provides a description of professional tasks for each item, ranging from beginner to expert level.

**Table 5:** Subscale ‘Critical nursing procedures’ in job domain 1 ‘Nursing & Patient Care,’ with the item and the corresponding answer category descriptions of professional tasks to assess the nurses’ professional performance level from beginner to expert.

Item	Performance levels	Description of professional tasks from beginner to expert level.
Decisions and nursing procedures	Beginner	In consultation with colleagues I can make decisions and proceed on the basis of the applicable standards and/or guidelines for the nursing process.
	Competent	I can make independent decisions and reflect on my work, evaluating my actions with colleagues to determine whether we are doing the right thing.
	Proficient	I reflect on my decisions and actions and evaluate them with colleagues to determine whether the standards and/or guidelines should be adapted.
	Expert	I monitor whether our work in the team follows the applicable criteria and set up meetings with everyone concerned to adjust decisions where necessary.
Recording and accountability of nursing procedures	Beginner	I can record my decisions and actions in standard situations and account for them according to the current standards and/or guidelines.
	Competent	In certain situations, I can deviate from the applicable standards and/or guidelines and provide reasons for doing so.
	Proficient	I also do so in complex situations: determining care needs for multimorbidity and/or urgent care; I know when to consult others.
	Expert	I encourage colleagues to consciously record and account for their actions, and support those who struggle with this.
Evaluation and reflection with patients and/or next-of-kin	Beginner	I hardly ever evaluate or reflect with patients on their experience of the care given.
	Competent	I evaluate and reflect with patients and/or next-of-kin on the care procedures and take action if individual patient satisfaction warrants it.
	Proficient	I can also do this in complex situations, if patients are not satisfied with the care actions.
	Expert	I discuss matters with the team if there is cause to improve the quality of evaluation and reflection with patients and/or next-of-kin.

Fig. 1 in the supplementary Appendix presents the timeline (2013-2023) for the iterative ECD of the FAN instrument, including the 93-item version presented in this manuscript.

#### 3.4.3 Evaluation of FAN with the Confirmatory Factor Analysis

Skewness and kurtosis were evaluated for each subscale, and the Shapiro test indicated that univariate normality was rejected across all subscales. The overall Kaiser-Meyer-Olkin (KMO) value for the data was .98. The second-order Confirmatory Factor Analyses (CFA) of the three job domains, referred to as the three-factor model, yielded results with 13 subscales loading >.50, six >.40, and two >.30. For the overarching job domain, called the one-factor model, nine subscales loaded >.50, ten >.40, and two >.30, as shown in Table 6. When comparing the one-factor model to the three-factor model, the former was rejected, confirmed through a weighted least-squares approach. The likelihood ratio test produced a chi-square statistic of 3026.7 with 3 degrees of freedom and a p-value < 0.0001, indicating the rejection of the one-factor model. This partially supports assigning the subscales to the three job domains. Inter-domain correlations in the three-factor model were very high (around .90), indicating strong correlations among the three job domains, as shown in Table 6.

**Table 6:** Loadings from Confirmatory Factor Analysis for one-factor and three-factors (i.e., subscales distributed across three job domains: JD1, JD2, and JD3), abbreviations, estimation, and, per subscale, the lower and upper confidence intervals (CI), including correlations among the three factors with their lower and upper confidence intervals (CI).

	One factor loadings (JD1)			Three factor loadings (JD1, 2 and3)		
Job domains, subscales and abbriviations	Estimate	CI. lower	CI. upper	Estimate	CI. lower	CI. upper
JD1 Nursing process (NPR)	0.48	0.47	0.50	0.52	0.50	0.53
JD1 Technical skills and risk-awareness (TSR)	0.45	0.44	0.46	0.48	0.47	0.50
JD1 Safety and prevention (SPR)	0.35	0.34	0.36	0.37	0.36	0.38
JD1 Medical knowledge (MKL)	0.55	0.54	0.56	0.58	0.57	0.60
JD1 Identifying urgency of care (IUC)	0.47	0.46	0.49	0.50	0.49	0.51
JD1 Critical nursing procedures (CNP)	0.53	0.52	0.54	0.54	0.53	0.56
JD1 Patient or client focus (PCF)	0.42	0.41	0.43	0.43	0.42	0.44
JD1 Service in healthcare (SHC)	0.48	o.47	0.49	0.48	0.47	0.49
JD1 ICT skills and eHealth (ICT)	0.32	0.31	0.34	0.30	0.28	0.31
JD2 Information processing (IPR)	0.50	0.49	0.51	0.50	0.49	0.52
JD2 Communication techniques and skills (CTS)	0.51	0.50	0.53	0.53	0.52	0.54
JD2 Patient-safe communication (PSC)	0.49	0.48	0.51	0.51	0.50	0.52
JD2 Collaboration (inter-/multidisciplinary) (CBR)	0.53	0.52	0.54	0.54	0.53	0.55
JD2 Compassion and empathy (CEP)	0.41	0.40	0.43	0.42	0.41	0.43
JD2 Organizational process of care (OPC)	0.50	0.49	0.52	0.51	0.50	0.52
JD3 Coordination of care (CDC)	0.52	0.50	0.53	0.52	0.51	0.53
JD3 Patient safety culture (PSC)	0.53	0.52	0.54	0.55	0.54	0.56
JD3 Quality of care, (QCR)	0.53	0.52	0.54	0.56	0.55	0.57
JD3 Social action (SAT)	0.44	0.43	0.46	0.47	0.46	0.48
JD3 Knowledge and science (KSC)	0.40	0.38	0.41	0.44	0.43	0.46
JD3 Professionalism (PFS)	0.49	0.48	0.50	0.51	0.50	0.52
**Job domain correlations**				Estimate	CI. lower	CI. upper
JD1 ‘Nursing & Patient Care’ and JD2 ‘Collaboration & Communication’				0.92	0.91	0.92
JD1 ‘Nursing & Patient Care’ and JD3 ‘Managing & Improving’				0.87	0.86	0.88
JD2 ‘Communication & Collaboration’ and JD3 ‘Managing & Improving’				0.95	0.95	0.96

The three-factor structure demonstrated a Robust Comparative Fit Index of 0.93 and a Robust Tucker-Lewis Index of 0.92, both above 0.90, indicating a good fit. The Robust Root Mean Square Error of Approximation was .88, with a 90% confidence interval between .87 and .90, which is considered a reasonable fit, and the Standardized Root Mean Square was 0.04, indicating a good fit (Goretzko et al., 2024; Sathyanarayana & Mohanasundaram, 2024). The one-factor structure showed a Robust Comparative Fit Index of 0.88 and a Robust Tucker-Lewis Index of 0.87, both acceptable. The Robust Root Mean Square Error of Approximation was 0.12, with a 90% confidence interval between 0.11 and 0.12, regarded as a reasonable fit, and the Standardized Root Mean Square was 0.05, indicating a good fit (Goretzko et al., 2024; Sathyanarayana & Mohanasundaram, 2024).

**Fig. 2:**
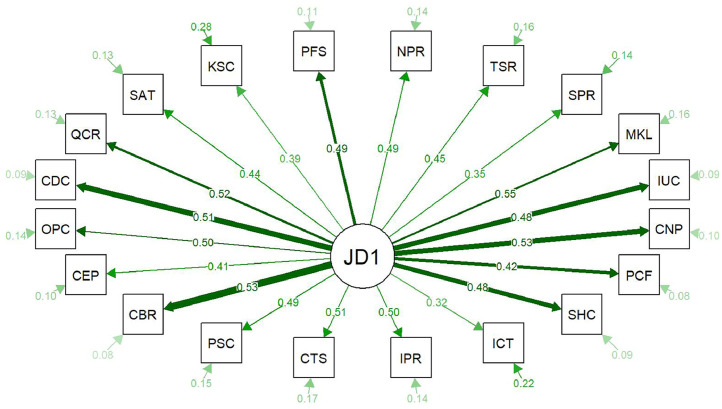
Confirmatory Factor Analysis plot for a one-factor model (i.e., all subscales included within one overarching job domain: JD1), displaying factor loadings, factor correlations, and error variances for each subscale, with abbreviated subscale names. Thicker green lines indicate higher loadings. The meanings of the abbreviations are provided in Table 6.

**Fig. 3:**
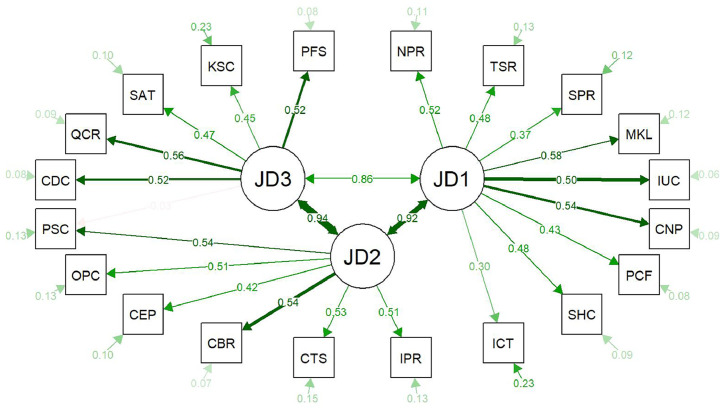
Confirmatory Factor Analysis plot of three factors (i.e., subscales distributed across three job domains: JD1, JD2, and JD3), showing factor loadings, factor correlations, and error variances between the job domains and the underlying subscales within each job domain, with abbreviated subscale names. Thicker green lines indicate higher loadings. Abbreviations are defined in Table 6.

Fig. 2, Fig. 3 show the factor loadings, correlations among factors, and error variances from the two CFA analyses, with subscale names abbreviated in square boxes. Larger loadings are represented by thicker lines. The abbreviations are explained in Table 6.

#### 3.4.4 Evidence of expected differences in nurses’ performance based on educational level

The independent-samples t-test across the three job domains for the two nursing educational levels indicated that, on average, bachelor-educated nurses performed at a higher level than vocationally educated nurses (see Table 7), with a medium effect size according to Cohen’s (1988) criteria.

**Table 7:** Mean (M) and standard deviation (SD) of vocational (n = 3,286) and bachelor (n = 2,051) educated nurses’ performance levels, with independent-samples t-test and Cohen’s d per job domain JD1, JD2 and JD3. Scoring range: 1, beginner; 2, competent; 3, proficient; and 4, expert.

	Vocational educated nurses (n = 3,286)		Bachelor educated nurses (n = 2,051)		Independent-samples *t*-test *df*(5,335)		Cohen’s d
	M	SD	M	SD	*T*	P*	*d*
JD1: Nursing & Patient Care	2.7	0.51	2.8	0.48	-6.96	<.001	0.50
JD2: Collaboration & Communication	2.7	0.54	2.8	0.52	-7.23	<.001	0.53
JD3: Managing & Improving	2.3	0.53	2.5	0.53	-12.81	<.001	0.53

⁎ P two-sided significance p <.001

The average performance level of nurses with vocational (n=3,286) and bachelor’s (n=2,052) education was lower in job domain 3, Managing & Improving, compared to job domains 1, Nursing & Patient Care, and 2, Collaboration & Communication. No difference was observed between job domains 1 and 2. This pattern indicates that job domain 3, Managing & Improving, involves more complex professional tasks than the other two domains.

**Fig. 4:**
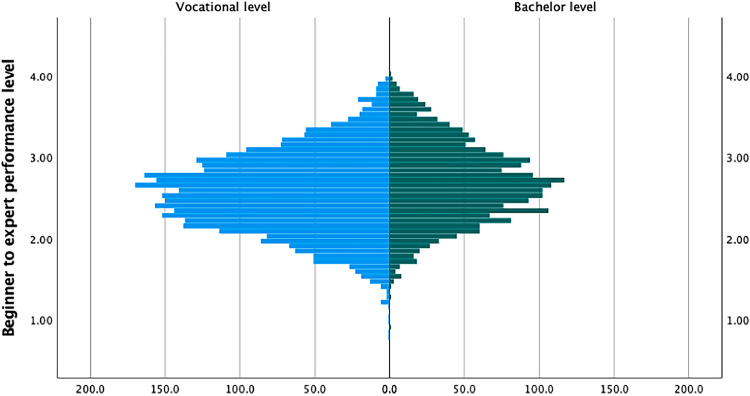
Population pyramid histogram showing the frequency distribution of four performance levels (1) beginner, (2) competent, (3) proficient, and (4) expert) on the vertical axis, for vocational (n=3286) and bachelor’s (n=2051) educated registered nurses. This data is within the total Formative Assessment for Nurses' Professional Performance (FAN) instrument, which includes all 21 subscales and 93 items.

Nurses with a bachelor’s degree perform slightly higher than registered nurses with vocational education, and relatively few nurses reach the beginner or expert level. It is shown in Figure 4.

#### 3.4.5 Evidence of expected variations in nurses’ performance according to their job roles

One-way ANOVA showed differences in performance levels on the total FAN among general nurses (n=2,946), specialized nurses (n=1,764), and senior nurses (n=403) (F(5,5404) = 108.930; p < .001) among those with a bachelor’s degree or a vocational background. On average, senior nurses’ performance level was the highest (M = 2.9; SD = 0.50), whereas general nurses’ was the lowest (M = 2.5; SD = 0.46). Specialized nurses scored in between (M = 2.7; SD = 0.48). Post hoc pairwise comparisons with Tamhane P-values revealed a statistically significant difference (p < .001) in performance between general nurses and both specialized and senior nurses. Additionally, senior nurses scored statistically significantly higher than specialized nurses (p < .001), with an Eta-squared effect size of .092, indicating a medium effect. Complete details are provided in the supplementary Appendix, Tables 3a, 3b, and 3c.

#### 3.4.6 Evidence of expected differences in nurses’ workplace performance

One-way ANOVA showed a statistically significant difference in mean total FAN scores among various nursing groups. Nurses in ‘Acute care,’ which includes the Intensive Care Unit, Emergency Room, and Cardiac Care Unit (n=485), had a higher overall mean score (M=2.8; SD=0.52) than those in ‘Surgical care’ (n=1,195), ‘Medical care’ (n=1,716), ‘Woman-mother-child care’ (n=802), ‘Dialysis care’ (n=171), and ‘Rehabilitation care’ (n=118). The mean FAN scores for ‘Acute care’ nurses were significantly higher than those for ‘Surgical care’ and ‘Medical care’ nurses (M=2.6; SD=0.47 and M=2.6; SD=0.48, respectively), with F(13,5397) = 16.190 and p < .001. Post hoc pairwise comparisons using Tamhane P-values across all job roles revealed a statistically significant difference (p < .001) in performance levels between nurses in ‘Acute care’ and the other five groups. The performance level of ‘Acute care’ nurses was statistically significantly higher than that of nurses in the other settings (p < .001). The effect size, measured by Eta-squared, was .038, indicating a small effect. Complete details are available in Tables 4a, 4b, and 4c in the supplementary Appendix.

#### 3.4.7 Nurses’ performance and their goals for professional development

The paired-samples analysis across the three job domains and 21 subscales related to nurses’ performance and goal levels showed that nurses’ self-stated goals were statistically significantly higher than their actual performance. See Table 8. Cohen’s *d* effect sizes ranged from .52 to .64, indicating a medium effect size (Cohen, 1988).

**Table 8:** Mean (M) and standard deviation (SD) of nurses’ performance levels and their professional development goals (n = 5,411), paired-samples *t*-test results, Cohen’s d, across three job domains, JD1, JD2, JD3, and 21 subscales. Scoring range: 1, beginner; 2, competent; 3, proficient; 4, expert.

	Performance level (N =5,411)		Goal (N = 5,411)		Paired-samples *t*-test *df*(5,410)		Cohen’s d
	M	SD	M	SD	*T*	P*	
**JD1: Nursing & Care**	2.7	0.50	3.1	0.59	-53.49	<.001	0.52
Nursing process	3.0	0.67	3.4	0.72	-44.56	<.001	0.63
Technical skills and risk awareness	2.8	0.67	3.1	0.70	-44.42	<.001	0.61
Safety and prevention	2.9	0.51	3.3	0.62	-39.19	<.001	0.57
Medical knowledge	2.8	0.70	3.3	0.71	-50.55	<.001	0.64
Identifying urgency of care	2.8	0.60	3.2	0.67	-50.19	<.001	0.58
Critical nurse procedures	2.8	0.65	3.3	0.71	-46.65	<.001	0.64
Patient or client focus	2.7	0.52	3.1	0.64	-47.42	<.001	0.58
Services in healthcare	2.7	0.69	3.0	0.79	-44.85	<.001	0.58
ICT skills and eHealth	2.0	0.63	2.4	0.79	-52.38	<.001	0.52
**JD2: Collaboration & Communication**	2.7	0.53	3.1	0.62	-55.85	<.001	0.54
Information processing	3.0	0.64	3.4	0.70	-39.95	<.001	0.64
Communication techniques and skills	2.8	0.69	3.2	0.74	-52.21	<.001	0.64
Patient-safe communication	2.6	0.63	3.1	0.65	-64.21	<.001	0.60
Collaboration (inter- and multidisciplinary)	2.7	0.64	3.1	0.72	-49.53	<.001	0.61
Compassion and empathy	2.7	0.55	3.1	0.69	-38.58	<.001	0.61
Organizational process of care	2.4	0.66	2.8	0.78	-50.82	<.001	0.59
**JD3: Managing & Improving**	2.4	0.54	2.9	0.69	-61.26	<.001	0.54
Coordination of care	2.6	0.63	3.0	0.72	-49.34	<.001	0.57
Patient safety culture	2.5	0.62	3.0	0.73	-54.56	<.001	0.61
Quality of Care	2.4	0.66	2.9	0.78	-59.79	<.001	0.61
Social action	2.3	0.61	2.7	0.79	-51.43	<.001	0.60
Knowledge and science	2.1	0.69	2.6	0.87	-64.14	<.001	0.61
Professionalism	2.5	0.60	3.0	0.72	-52.26	<.001	0.60

⁎ P two-sided significance p <.001

#### 3.4.8 Evaluation of the FAN along Mislevy’s ‘Eight Validity Challenges’

Mislevy’s (2016) ‘Eight Validity Challenges’ were used to evaluate how nurses’ capabilities are harnessed in the FAN instrument during ECD stages one through four. Seven of these ‘Eight Validity Challenges’ are integrated into the framework shown in Table 1 for developing the FAN. Validity Challenge five, ‘What is the role of measurement models?’, addresses the function of the FAN as a ‘Performance Assessment,’ ‘Needs Assessment,’ and ‘Assessment for Learning.’ Validity Challenge six, ‘How do we “score” complex, interactive performances at scale?’, is integrated into the framework and addressed after completing the assessment. This includes interacting with the team leader, colleagues, or educators involved to verify job performance; interpreting and using the assessment outcome; setting goals for professional development; and implementing educational interventions based on nurses’ needs. A detailed overview of this evaluation is provided in the supplementary Appendix, Table 5.

## 4 Discussion

This study developed and evaluated a Formative Assessment instrument for Nurses' Professional Performance using the Evidence-Centered Design Model by Mislevy (2012). It examined how nurse performance is harnessed from beginner to expert across the ‘Eight Validity Challenges’ described by Mislevy (2016). The instrument is formative and developmental, designed to assess authentic nurses’ tasks across a broad scope of the profession in various work settings at the behavioral level. All essential nurses’ tasks are represented in 21 subscales and 93 items, covering performance levels from beginner to expert. Initially, the seven CanMEDS domains served as an overarching framework; the FAN comprises three domains. Job domain 1, ‘Nursing & Patient Care,’ involves direct care and aligns with the CanMEDS domain of Nurse Expert. Job domain 2, ‘Collaboration & Communication,’ is from socio-cognitive and socio-constructive perspectives and aligns with the CanMEDS Collaboration Partner and Communicator domains. It addresses the interplay of cognitive processes within individual professionals’ interactions among nurses and their tasks in real-world contexts, as experienced by them, as well as among colleagues or disciplines related to social practices, including Linguistic, Cultural, and Substantive (LCS) patterns (Mislevy, 2016; Filliettaz, 2014; Vygotsky, 1978). Job domain 3 appears to be more complex within the broad scope of nurses’ work, aligning with the CanMEDS domains of Reflective ‘EBP’ professional, Health Promoter, Organizer/Leader, and Quality Promoter.

The items were validated through several rounds of verification and an Item Content Validity survey. Whereof, a number of FAN items are designed with the future of nursing in mind, such as those in the subscale ‘ICT skills and eHealth.’ For example, the two items “Use of ICT for remote care (telehealth) and Patients' self-monitoring and self-diagnosis.” Currently, a higher percentage of scores fall at a lower performance level compared to other items in the FAN administered by the same nurse. As technology advances in nursing, these results are expected to improve. It is believed that performance on this subscale will develop accordingly unless these tasks are delegated only to a few nurse experts or other professionals within an organization. Given that the nurses–patient ratio and universal health coverage will be strained by aging populations and a nurse shortage, these tasks are expected to be assigned to a larger group of nurses. Therefore, the development process continues with ongoing validation as the nursing field evolves.

The instrument's validity, reliability, and feasibility were assessed using empirical data from a large sample of nurses across 22 hospitals and two rehabilitation centers. It demonstrated strong evidence through the General Partial Credit Model and Confirmatory Factor Analysis (CFA). The CFA results indicated either three factors (representing three job domains) or a single overarching factor (representing one comprehensive job domain). The FAN was confirmed in both scenarios, given the large sample size (n=5,411) and strong correlations among the three job domains. Additionally, all 21 subscales were retained, either as a single factor (one main job domain) or divided into three factors (three job domains), to represent nurses’ work and professional performance across various tasks in different workplace settings. The strong correlations among the three job domains support the view that it follows a holistic Whole-Task approach, as described in the ‘Four-Component Instructional Design’ (4C/ID) Model by Van Merriënboer & Kirschner (2018). The overarching factor covers the broad nurses’ job domain, while the three factors specify particular areas, each with underlying subscales and items. Using a single, comprehensive job domain instead of dividing it into three areas helps prevent compartmentalization, fragmentation, and the transfer paradox.

Additionally, evidence shows that the instrument distinguishes between two educational levels, six job roles, and various workplace settings. It also highlights nurses’ professional development goals. Consequently, nurses’ needs for advancement to the next performance level can be recognized.

The finding that relatively few nurses reached the expert level (Figure 4) can be explained by the lengthy development period required to attain this level (Ericsson, 2018). Expert level is reflected in professional task descriptions that demonstrate nurse leadership from a metacognitive perspective, including synthesis and advanced problem-solving skills to undertake proactive action. This aligns with research showing that influential nurse leaders are essential as change agents and role models (Hakvoort et al., 2022; Wei et al., 2023). In our study, nearly half of the participating nurses had less than five years of clinical experience in the department. A relatively small number of nurses were at the beginner level of expertise, as only registered nurses participated in this study.

The recognition that nurses’ work involves physical, emotional, cognitive, and organizational aspects (Jackson et al., 2021) is reflected in the most widely used competence assessments and is a key part of the FAN assessment instrument. The main differences between the most common competence assessments for nurses’ performance and the FAN instrument are: 1) the assessment method, focusing on competence versus performance; 2) the structure, with item-level competence statements versus performance levels answer categories with task descriptions from beginner to expert—a cumulative scale; 3) the level of detail, or how thoroughly nurses’ competence or performance is evaluated; and 4) each task description, from beginner to expert, not only indicates performance but also helps guide nurse professionals in developing their expertise.

Overall, valid measures can be applied across and within groups in various workplaces, depending on the measurement's purpose.

### 4.1 Strengths and Limitations

This study used convenience sampling, including samples from experienced experts, which may be a limitation. However, many experienced nurses and other professionals participated at different stages of the instrument's development, providing a diverse range of perspectives.

A strength of the study is its broad inclusion of nurses at various stages of development. The assessment instrument reflects the wide range of their professional tasks, as performed and evaluated across different roles, educational backgrounds, and workplaces. In this study, important aspects of validity, reliability, and feasibility have been examined, but additional validity research could be useful, such as collecting more evidence of discriminant validity (distinguishing it from other target groups) and generalizability.

The data were used formatively in meetings between team leaders and nurses for professional and career development. This could be a constraint influencing participants' responses. Regarding response bias, nurses offered a range of responses across the items, from beginner to expert levels. At the individual nurse level, the validity of the item-level responses was verified during face-to-face developmental meetings organized for this purpose. These meetings involved providing examples of performance in the work environment at the indicated level. Respondents were aware in advance that the level they indicated should match what they typically demonstrate in practice. The results show that some subscales, such as ‘ICT skills and eHealth’ and ‘Knowledge and Science,’ scored relatively lower than others. These findings reflect the accuracy of completing the FAN in the workplace and its alignment with the appropriate performance level. Additionally, the willingness to set a specific professional development goal indicates motivation to complete it successfully. Furthermore, the independent-samples t-test and ANOVA reveal differences among nurses based on educational level, job role, and workplace setting, as expected.

A strength of the extended development timeline from 2013 to 2023 is that it has refined and matured the FAN through 10 iterations, including subsequent implementations and evaluations of its overall usefulness and the completeness of the entire project. A limitation might be the environmental changes nurses experience during this period, which could have altered tasks, impacted their work, and, as a result, affected performance across participants over time. However, the evidence showed that the validity and generalizability were maintained.

The data collected from the initial 77-item version to the current 93-item version includes various implementations of programs and projects in hospitals and rehabilitation centers aimed at measuring nurses’ performance, professional development needs, and career advancement, including distinctions in job roles. The assessment instrument has proven widely applicable across academic institutions, leading clinical teaching hospitals, general hospitals, and rehabilitation centers, covering diverse workplace settings in the Netherlands. Throughout this timeline, the versatility and usefulness of the performance assessment for nurses are also demonstrated in other healthcare settings, such as nursing homes, home care, and mental health facilities, with slight adjustments, though data from these settings are not included in this study. This is a limitation for the validity of the FAN across healthcare organizations other than hospitals and rehabilitation centers.

Although the instrument was developed in the Netherlands and may therefore reflect aspects of a different system and culture, the care receiver—that is, the human being—is universal.

### 4.2 Implications for Practice

Our findings indicate that the assessment instrument reflects how a professional nurse performs, highlighting the overall development of performance across various tasks. It captures nurses' growth in expertise throughout the full range of nurses’ responsibilities. The ‘Whole-Task’ approach in the instrument helps prevent compartmentalization, fragmentation, and the transfer paradox during nurses’ professional growth by engaging with meaningful ‘Whole Tasks’ in the workplace (Van Merriënboer & Kirschner, 2018; Francom & Gardner, 2014; Francom, 2018). The assessment can identify gaps and direct professional development and training. This is especially crucial when nurses need to enhance their capacity, meet staffing demands, ensure performance aligns with nurse-patient ratios, and improve patient outcomes (Aiken et al., 2002; Needleman et al., 2011; Aiken et al., 2016; Ball et al., 2018; Twigg et al., 2019).

Using the assessment tool for performance measurement can help unlock nurses’ potential across various tasks. This instrument can be utilized for self-assessment to support learning and identify areas for improvement. Its outcomes can guide goal-setting or facilitate discussions with team leaders, colleagues, and others involved in professional development to boost expertise and, at the same time, enhance the quality of care. As the quality of care depends on performance. Additionally, it can incorporate performance levels into capacity management and strategic staffing planning (Tate et al., 2024).

### 4.3 Recommendations for Further Research

Further research is needed to clarify how nurses’ task-related competencies and performance standards can be interpreted to support career development. This could speed up development and readiness for practice, especially as work environments evolve and mobility between jobs increases, including for pre-registration and undergraduate nurses. Additionally, a follow-up study could examine how performance levels can be used at the organizational level for strategic nurse planning and management. This involves staffing, skills mix, nurse-patient ratios, and reducing missed nursing care, which can improve nurses’ well-being and patient outcomes. To our knowledge, registered nurses’ performance levels are often overlooked as a key factor in policy and practice decisions. Recognizing this alongside educational background and years of workplace experience could provide valuable statistical insights. For instance, considering nurses’ performance levels when setting nurse-patient ratios could improve skill matching and prevent mismatches between expertise and workload. Moreover, the assessment instrument offers opportunities to investigate relationships among nurses’ performance levels, alignment of organizational and nurses’ goals, job embeddedness, turnover intentions, patient outcomes (Wang et al., 2024), and nurses’ engagement with the workplace. This enables a deeper understanding of competence and performance (While, 1994) and supports research into how competence development affects professional performance by aligning competence assessment subscales or items with (parts of) the FAN. Future research should also evaluate whether the instrument's results are generalizable across different cultures.

## 5 Conclusions

This study developed and evaluated the Formative Assessment instrument for Nurses’ Professional Performance (FAN), designed to measure performance from beginner to expert across the broad spectrum of nurses’ tasks in practice. The study provided evidence supporting the construct validity of the assessment instrument as a comprehensive ‘Whole Task’ framework, which intends to encompass the nurses’ job domain. This domain is divided into three specific areas, with 21 subscales, 93 items, and four answer categories for each item, ranging from beginner to expert. It includes professional task descriptions at the behavior level, covering constituent knowledge, skills, and attitudes across these four performance levels. The estimated reliability, factor loadings, discrimination parameters, and the monotonicity of answer category thresholds align with current psychometric standards. Confirmatory factor analysis supports a single overarching job domain. The instrument demonstrates validity, reliability, and feasibility for assessing performance across the broad scope of nurses’ work at both vocational and bachelor’s levels, in various roles and workplace settings. Given the strong interest in nurses' career development, the instrument can provide constructive feedback to help close gaps among current expertise, goals, and the new requirements of future roles. Additionally, it can inform strategic decisions regarding nurses’ professional growth and its acceleration. This is vital for building a healthcare workforce capable of meeting increasingly complex population health needs, aligning with the skills and capabilities required in the health sector (World Health Organization, 2025), with nurses’ performance at its core.

## Copyright © 2026 Freda M.D. Vasse. All rights reserved

The Formative Assessment for Nurses’ Professional Performance (FAN) is the intellectual property of Freda M.D. Vasse, who retains all proprietary rights, including translation, adaptation, and use. The instrument is exclusively licensed to Frenetti® for use and distribution within its software platform for measurement, learning and optimization.

## CRediT authorship contribution statement

**Freda M.D. Vasse:** Writing – original draft, Visualization, Validation, Software, Resources, Project administration, Methodology, Investigation, Formal analysis, Data curation, Conceptualization. **Wim P. Krijnen:** Writing – review & editing, Methodology, Formal analysis. **Marieke F. Van Der Schaaf:** Writing – review & editing, Supervision. **Evelyn J. Finnema:** Writing – review & editing, Supervision.

## Declaration of competing interest

The authors declare the following financial interests/personal relationships, which may be considered as potential competing interests:

The author, F.M.D. Vasse is the founder, CEO, and owner of Frenetti® to which the instrument is exclusively licensed for the implementation and distribution of the Formative Assessment for Nurses' Professional Performance (FAN), representing a potential financial conflict of interest. This relationship has been disclosed to all co-authors and the affiliated academic institution. Research collaborations and access to the instrument for scientific purposes are available upon reasonable request. No external funding was received for the development, evaluation, or publication of this research.

## Appendix Supplementary materials

Image, application 1

Image, application 2

Image, application 3

Image, application 4

Image, application 5

Image, application 6
