# Supplementary material for: Nurses’ Professional Performance: The Development and Evaluation of a Formative Workplace-Based Self-Assessment Instrument
Source: Int J Nurs Stud Adv. 2026 May 14;10:100542. doi: 10.1016/j.ijnsa.2026.100542 (PMC13196436; doi:10.1016/j.ijnsa.2026.100542)
Supplement: Supplementary file 1 [file mmc1.pptx]

## Slide 1
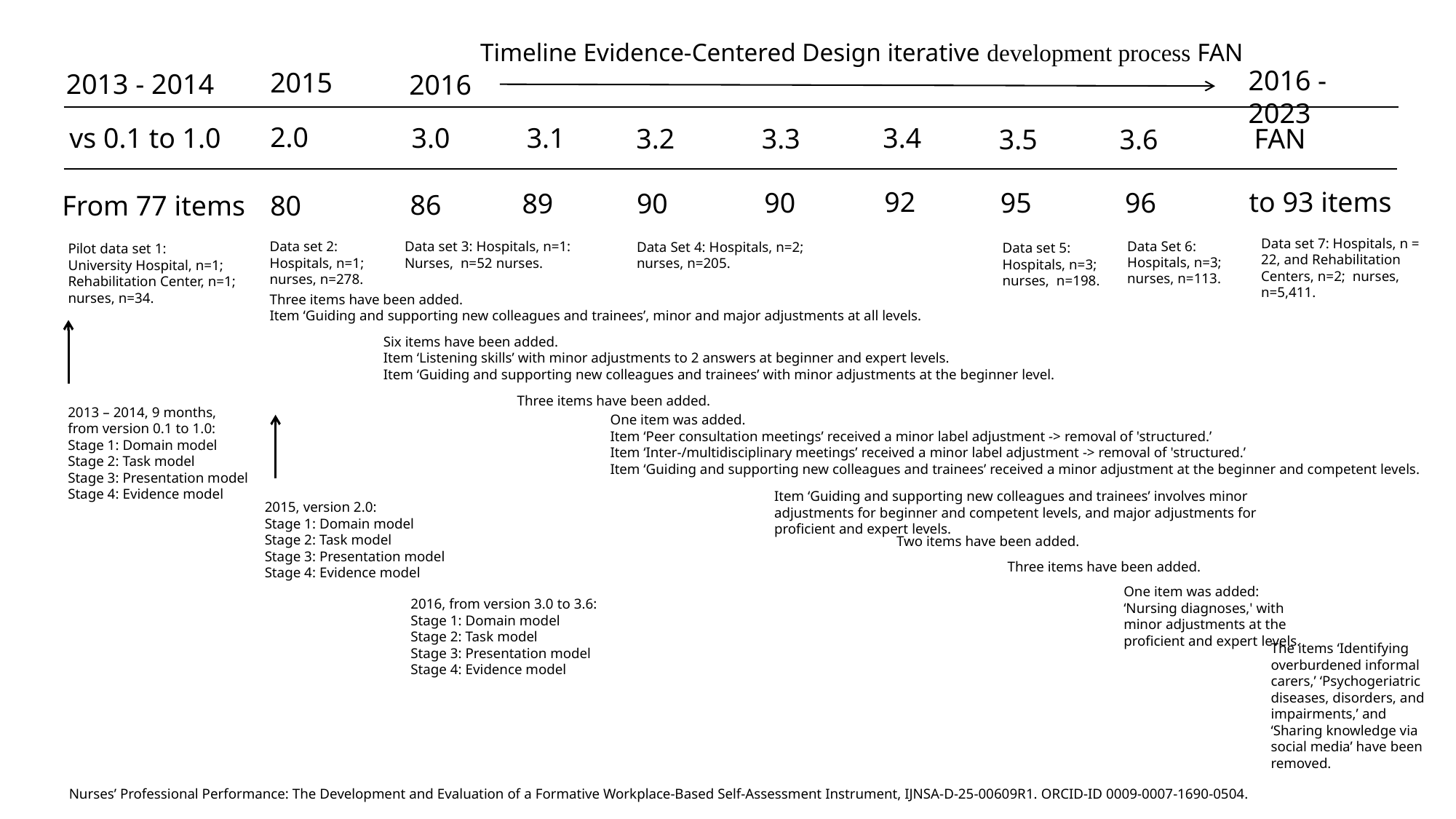

Timeline Evidence-Centered Design iterative development process FAN
2016 - 2023
2015
2013 - 2014
2016
2.0
3.0
3.4
3.1
vs 0.1 to 1.0
3.3
FAN
3.2
3.5
3.6
to 93 items
92
95
96
90
89
90
86
From 77 items
80
Data set 7: Hospitals, n = 22, and Rehabilitation Centers, n=2; nurses, n=5,411.
Data Set 6: Hospitals, n=3; nurses, n=113.
Data set 3: Hospitals, n=1:
Nurses, n=52 nurses.
Data set 2: Hospitals, n=1; nurses, n=278.
Data Set 4: Hospitals, n=2; nurses, n=205.
Data set 5: Hospitals, n=3; nurses, n=198.
Pilot data set 1:
University Hospital, n=1; Rehabilitation Center, n=1; nurses, n=34.
Three items have been added.
Item ‘Guiding and supporting new colleagues and trainees’, minor and major adjustments at all levels.
Six items have been added.
Item ‘Listening skills’ with minor adjustments to 2 answers at beginner and expert levels.
Item ‘Guiding and supporting new colleagues and trainees’ with minor adjustments at the beginner level.
Three items have been added.
2013 – 2014, 9 months,
from version 0.1 to 1.0:
Stage 1: Domain model
Stage 2: Task model
Stage 3: Presentation model
Stage 4: Evidence model
One item was added.
Item ‘Peer consultation meetings’ received a minor label adjustment -> removal of 'structured.’
Item ‘Inter-/multidisciplinary meetings’ received a minor label adjustment -> removal of 'structured.’
Item ‘Guiding and supporting new colleagues and trainees’ received a minor adjustment at the beginner and competent levels.
Item ‘Guiding and supporting new colleagues and trainees’ involves minor adjustments for beginner and competent levels, and major adjustments for proficient and expert levels.
2015, version 2.0:
Stage 1: Domain model
Stage 2: Task model
Stage 3: Presentation model
Stage 4: Evidence model
Two items have been added.
Three items have been added.
One item was added: ‘Nursing diagnoses,' with minor adjustments at the proficient and expert levels.
2016, from version 3.0 to 3.6:
Stage 1: Domain model
Stage 2: Task model
Stage 3: Presentation model
Stage 4: Evidence model
The items ‘Identifying overburdened informal carers,’ ‘Psychogeriatric diseases, disorders, and impairments,’ and ‘Sharing knowledge via social media’ have been removed.
Nurses’ Professional Performance: The Development and Evaluation of a Formative Workplace-Based Self-Assessment Instrument, IJNSA-D-25-00609R1. ORCID-ID 0009-0007-1690-0504.
