# Supplementary material for: Nurses’ Professional Performance: The Development and Evaluation of a Formative Workplace-Based Self-Assessment Instrument
Source: Int J Nurs Stud Adv. 2026 May 14;10:100542. doi: 10.1016/j.ijnsa.2026.100542 (PMC13196436; doi:10.1016/j.ijnsa.2026.100542)
Supplement: Supplementary file 2 [file mmc2.docx]

**Appendix, Table 1. ‘Action verbs’ usage in the FAN.**

Table 1. The number of ‘action verbs’ usage, subtotal, and total per category from the *revised* Bloom’s taxonomy (Anderson & Krathwohl, 2001) in the task descriptions from beginner to expert level per job domain (JD)1, 2, and 3, with examples of ‘action verbs’.

| JD | Category | Examples of ‘action verbs’ | Beginnerer | Competent | Subtotal | Proficient | Expert | Subtotal |
| --- | --- | --- | --- | --- | --- | --- | --- | --- |
| JD1 | I Remembering | Find, which, when, where | 20 | 16 | 36 | 7 | 9 | 16 |
|  | II Understanding |  | 0 | 0 | 0 | 0 | 0 | 0 |
|  | III Applying | Apply, utilize, identify | 8 | 9 | 17 | 2 | 10 | 12 |
|  | Subtotal |  | 28 | 25 | **53** | 9 | 19 | **28** |
|  | IV Analyzing | Analyze, distinguish | 2 | 2 | 4 | 5 | 2 | 7 |
|  | V Evaluation | Support, evaluate, assess | 8 | 8 | 16 | 15 | 12 | 27 |
|  | VI Creating | Discuss, adjust, improve | 0 | 1 | 1 | 4 | 24 | 28 |
|  | Subtotal |  | 10 | 11 | **21** | 24 | 38 | **62** |
| JD2 | I Remembering | Find, where, when, name | 8 | 4 | 12 | 2 | 0 | 2 |
|  | II Understanding | Demonstrate | 1 | 1 | 2 | 0 | 0 | 0 |
|  | III Applying | Identify, develop, identify | 2 | 1 | 3 | 2 | 4 | 6 |
|  | Subtotal |  | 11 | 6 | **17** | 4 | 4 | **8** |
|  | IV Analyzing | Analyze, integrate | 0 | 0 | 0 | 2 | 0 | 2 |
|  | V Evaluation | Support,evaluate,determine | 2 | 3 | 5 | 7 | 9 | 16 |
|  | VI Creating | Discuss, adjust, propose | 1 | 1 | 2 | 5 | 11 | 16 |
|  | Subtotal |  | 3 | 4 | **7** | 14 | 20 | **34** |
| JD3 | I Remembering | Find, where, what, which | 5 | 5 | 10 | 8 | 5 | 13 |
|  | II Understanding | Translate | 0 | 0 | 0 | 1 | 0 | 1 |
|  | III Applying | Utilize, plan, solve | 2 | 5 | 7 | 6 | 4 | 10 |
|  | Subtotal |  | 7 | 10 | **17** | 15 | 9 | **24** |
|  | IV Analyzing | Take part in, determine | 1 | 2 | 3 | 3 | 2 | 5 |
|  | V Evaluation | Support, evaluate, assess | 2 | 4 | 6 | 13 | 15 | 28 |
|  | VI Creating | Discuss, propose, improve | 3 | 4 | 7 | 10 | 13 | 23 |
|  | Subtotal |  | 6 | 10 | **16** | 26 | 30 | **56** |
|  | Total category ‘lower-order thinking skills’ I, II, III | | 46 | 41 | **87** | 28 | 32 | **60** |
|  | Total category ‘higher-order thinking skills’ IV,V, VI | | 19 | 25 | **44** | 64 | 88 | **152** |
