# Supplementary material for: Nurses’ Professional Performance: The Development and Evaluation of a Formative Workplace-Based Self-Assessment Instrument
Source: Int J Nurs Stud Adv. 2026 May 14;10:100542. doi: 10.1016/j.ijnsa.2026.100542 (PMC13196436; doi:10.1016/j.ijnsa.2026.100542)
Supplement: Supplementary file 3 [file mmc3.docx]

**Appendix, Table 2. General Partial Credit Model.**

| Table 2. *Psychometric properties over* Formative Assessment for Nurses' Professional Performance *instrument’s 3 job domains, 21 subscales and 93 items with percentages per category of response for each performance level, Cronbach’s alpha* *if deleted, Factor loadings, General Partical Credit Model coefficients and Discrete parameter obtained with the Generalized Partial Credit Model test.* | | | | | | | | | | |
| --- | --- | --- | --- | --- | --- | --- | --- | --- | --- | --- |
| **Subscales** | **Percentages per category of response** | | | | **α if**  **deleted** | **Factor loadings** | **Generalized Partial Credit Model coefficients** | | | **Discr. parameter** |
| **Job domain 1: Nursing & Care** | **Beginner level** | **Competent level** | **Proficient level** | **Expert level** | **Cron.**  **alpha** | **Loadings** | **Expertise level 1/2** | **Expertise level 2 /3** | **Expertise**  **level 3/4** | **Discr** |
| **Nursing process** |  |  |  |  |  |  |  |  |  |  |
| Principles of the nursing process | 5 | 19 | 63 | 13 | .88 | 0.67 | -2.03 | -1.00 | 1.53 | 1.79 |
| Admission, care needs and intervention | 2 | 12 | 41 | 45 | .87 | 0.79 | -2.40 | -1.27 | 0.14 | 2.75 |
| Drawing up nursing care plans | 3 | 18 | 52 | 27 | .86 | 0.83 | -2.11 | -0.91 | 0.69 | 3.57 |
| Nursing diagnoses | 3 | 17 | 49 | 32 | .86 | 0.83 | -2.13 | -0.95 | 0.54 | 3.42 |
| Outcome of nursing care plan | 1 | 20 | 47 | 32 | .87 | 0.77 | -2.92 | -0.94 | 0.56 | 2.54 |
| Hand-off and reporting procedures | 2 | 27 | 41 | 30 | .88 | 0.69 | -2.72 | -0.65 | 0.64 | 1.61 |
| *Cronbach’s alpha total subscale* |  |  |  |  | .89 |  |  |  |  |  |
| **Technical skills and risk awareness** |  |  |  |  |  |  |  |  |  |  |
| Reserved and high-risk procedures | 5 | 20 | 61 | 14 | .58 | 0.79 | -1.96 | -0.85 | 1.27 | 2.57 |
| Technical nursing skills and procedures | 6 | 35 | 30 | 29 | .66 | 0.71 | -2.08 | -0.17 | 0.53 | 1.86 |
| Medical devices and support equipment | 4 | 20 | 73 | 4 | .70 | 0.64 | -2.22 | -1.02 | 2.45 | 1.92 |
| *Cronbach’s alpha total subscale* |  |  |  |  | .74 |  |  |  |  |  |
| **Safety and prevention** |  |  |  |  |  |  |  |  |  |  |
| Hygiene regulations for prevention | 0 | 10 | 30 | 60 | .73 | 0.57 | -4.21 | -1.66 | -0.57 | 1.28 |
| Addressing my own safety and that of others | 0 | 32 | 59 | 8 | .69 | 0.68 | -3.97 | -0.57 | 1.76 | 2.19 |
| Dealing with hospital waste | 2 | 41 | 52 | 5 | .67 | 0.71 | -2.56 | -0.21 | 2.04 | 2.32 |
| Working hygienically when providing ADL (Activity of Daily Life) care | 0 | 17 | 61 | 22 | .68 | 0.67 | -3.93 | -1.24 | 1.02 | 1.93 |
| *Cronbach’s alpha total subscale* |  |  |  |  | .75 |  |  |  |  |  |
| **Medical knowledge** |  |  |  |  |  |  |  |  |  |  |
| Anatomy – physiology | 3 | 18 | 45 | 34 | .78 | 0.83 | -2.06 | -0.91 | 0.44 | 3.35 |
| Somatic diseases, disorders and impairments | 3 | 17 | 51 | 29 | .78 | 0.85 | -2.11 | -0.95 | 0.56 | 4.09 |
| Pharmacology - Pharmacotherapy | 5 | 28 | 48 | 19 | .78 | 0.79 | -1.96 | -0.53 | 1.01 | 2.70 |
| Psychiatric diseases, disorders and impairments | 16 | 32 | 36 | 16 | .86 | 0.60 | -1.34 | -0.16 | 1.41 | 1.03 |
| *Cronbach’s alpha total subscale* |  |  |  |  | .84 |  |  |  |  |  |
| **Identifying urgency of care** |  |  |  |  |  |  |  |  |  |  |
| Recognizing symptoms | 2 | 19 | 63 | 16 | .79 | 0.85 | -2.26 | -0.87 | 1.05 | 4.85 |
| Identifying complications | 2 | 20 | 61 | 17 | .79 | 0.85 | -2.18 | -0.82 | 1.04 | 4.71 |
| Clinical reasoning | 3 | 22 | 61 | 14 | .81 | 0.74 | -2.24 | -0.85 | 1.34 | 2.32 |
| Unexpected and unstable situations (crisis intervention) | 1 | 18 | 72 | 9 | .83 | 0.65 | -3.00 | -1.16 | 1.78 | 2.01 |
| Supervision and support with psychosomatics | 14 | 24 | 42 | 20 | .85 | 0.65 | -1.25 | -0.56 | 1.17 | 1.21 |
| *Cronbach’s alpha total subscale* |  |  |  |  | .85 |  |  |  |  |  |
| **Critical nurse procedures** |  |  |  |  |  |  |  |  |  |  |
| Decisions and nursing procedures | 2 | 32 | 53 | 13 | .71 | 0.76 | -2.4 | -0.48 | 1.35 | 2.67 |
| Recording and accountability of nursing procedures | 5 | 20 | 55 | 21 | .70 | 0.78 | -1.94 | -0.86 | 0.95 | 2.56 |
| Evaluation and reflection with patients and/or next-of-kin | 3 | 25 | 50 | 23 | .74 | 0.71 | -2.54 | -0.79 | 0.93 | 1.87 |
| *Cronbach’s alpha total subscale* |  |  |  |  | .79 |  |  |  |  |  |
| **Patient or client focus** |  |  |  |  |  |  |  |  |  |  |
| Hospitality | 0 | 33 | 57 | 10 | .71 | 0.73 | -3.7 | -0.54 | 1.53 | 2.49 |
| Relationship with the patient and/or next-of-kin | 0 | 34 | 45 | 21 | .71 | 0.74 | -3.46 | -0.48 | 0.97 | 2.34 |
| Patient instruction and information | 1 | 18 | 7 | 11 | .75 | 0.62 | -2.94 | -1.18 | 1.65 | 1.88 |
| Intercultural interaction | 7 | 49 | 4 | 4 | .74 | 0.66 | -2.06 | 0.19 | 2.31 | 1.72 |
| *Cronbach’s alpha total subscale* |  |  |  |  | .78 |  |  |  |  |  |
| **Services in healthcare** |  |  |  |  |  |  |  |  |  |  |
| Coordinating care with patients | 1 | 18 | 65 | 16 | .85 | 0.71 | -2.94 | -1.12 | 1.22 | 2.35 |
| Self-management support and interventions | 4 | 41 | 42 | 13 | .85 | 0.73 | -2.29 | -0.12 | 1.40 | 2.13 |
| Self-management support with the aid of informal carers | 4 | 33 | 52 | 11 | .84 | 0.77 | -2.11 | -0.4 | 1.46 | 2.61 |
| Palliative care (prevention and alleviation of suffering) | 10 | 25 | 47 | 19 | .86 | 0.65 | -1.65 | -0.69 | 1.27 | 1.30 |
| Encouraging relatives and informal carers to help | 3 | 27 | 52 | 19 | .84 | 0.76 | -2.33 | -0.66 | 1.07 | 2.36 |
| Eliciting help from relatives and informal carers | 2 | 27 | 58 | 13 | .84 | 0.78 | -2.35 | -0.63 | 1.32 | 2.94 |
| *Cronbach’s alpha total subscale* |  |  |  |  | .87 |  |  |  |  |  |
| **ICT skills and eHealth** |  |  |  |  |  |  |  |  |  |  |
| ICT (Information Communication Technology) skills and applications | 10 | 59 | 26 | 5 | .79 | 0.73 | -1.52 | 0.62 | 1.89 | 2.49 |
| Use of electronic health records (EHRs) | 3 | 34 | 52 | 12 | .80 | 0.65 | -2.58 | -0.46 | 1.57 | 1.85 |
| Use of ICT for remote care (telehealth) | 52 | 37 | 8 | 2 | .79 | 0.71 | 0.13 | 1.69 | 2.25 | 1.80 |
| Organization of remote patient care (telehealth) | 21 | 35 | 38 | 5 | .82 | 0.57 | -1.04 | 0.06 | 3.03 | 0.89 |
| Patients' self-monitoring and self-diagnosis | 46 | 29 | 22 | 3 | .80 | 0.63 | 0.22 | 0.75 | 2.94 | 1.04 |
| Use of computer programs | 7 | 45 | 37 | 11 | .79 | 0.73 | -1.78 | 0.07 | 1.45 | 2.39 |
| *Cronbach’s alpha total subscale* |  |  |  |  | .83 |  |  |  |  |  |
| *Cronbach’s alpha for the total job domain 1* |  |  |  |  | .93 |  |  |  |  |  |
| **Job domain 2: Collaboration & Communication** | | | | | | | | | | |
| **Information processing** |  |  |  |  |  |  |  |  |  |  |
| Acquiring information | 4 | 24 | 52 | 19 | .64 | 0.84 | -1.83 | -0.65 | 0.96 | 3.67 |
| Exchanging information | 1 | 20 | 59 | 20 | .67 | 0.80 | -2.5 | -0.92 | 0.97 | 3.32 |
| Listening skills | 1 | 20 | 35 | 44 | .80 | 0.60 | -3.61 | -1.02 | 0.02 | 1.20 |
| *Cronbach’s alpha total subscale* |  |  |  |  | .78 |  |  |  |  |  |
| **Communication techniques and skills** |  |  |  |  |  |  |  |  |  |  |
| Communication skills | 4 | 30 | 46 | 20 | .74 | 0.79 | -2.04 | -0.48 | 0.97 | 2.60 |
| Structuring conversations | 4 | 41 | 47 | 8 | .74 | 0.79 | -2.00 | -0.14 | 1.60 | 3.11 |
| Reaching the goals and/or consensus in conversations | 5 | 21 | 50 | 25 | .76 | 0.74 | -1.98 | -0.86 | 0.84 | 2.11 |
| *Cronbach’s alpha total subscale* |  |  |  |  | .82 |  |  |  |  |  |
| **Patient-safe communication** |  |  |  |  |  |  |  |  |  |  |
| Giving feedback | 8 | 41 | 37 | 14 | .77 | 0.80 | -1.60 | -0.02 | 1.25 | 2.78 |
| Receiving feedback | 6 | 28 | 49 | 17 | .79 | 0.76 | -1.81 | -0.53 | 1.13 | 2.43 |
| Calling colleagues to account | 9 | 55 | 33 | 3 | .80 | 0.73 | -1.67 | 0.43 | 2.36 | 2.28 |
| Opening up (near miss) incidents for discussion | 3 | 46 | 39 | 13 | .80 | 0.70 | -2.63 | -0.02 | 1.44 | 1.82 |
| *Cronbach’s alpha total subscale* |  |  |  |  | .83 |  |  |  |  |  |
| **Collaboration (inter- and multidisciplinar)** |  |  |  |  |  |  |  |  |  |  |
| Teamwork | 1 | 15 | 69 | 15 | .88 | 0.66 | -3.11 | -1.34 | 1.34 | 2.07 |
| Peer consultation meetings | 7 | 40 | 30 | 23 | .85 | 0.81 | -1.74 | -0.04 | 0.81 | 2.84 |
| Inter-/multidisciplinary meetings | 10 | 41 | 31 | 18 | .86 | 0.80 | -1.54 | 0.07 | 1.04 | 2.57 |
| Multidisciplinary collaboration | 0 | 14 | 47 | 38 | .88 | 0.64 | -4.72 | -1.46 | 0.38 | 1.51 |
| Leadership tasks | 8 | 36 | 43 | 13 | .87 | 0.70 | -1.82 | -0.19 | 1.5 | 1.62 |
| Dealing with moral dilemmas | 2 | 42 | 43 | 13 | .87 | 0.70 | -2.76 | -0.15 | 1.43 | 1.81 |
| Team meetings | 15 | 42 | 24 | 19 | .86 | 0.76 | -1.30 | 0.35 | 0.95 | 1.99 |
| *Cronbach’s alpha total subscale* |  |  |  |  | .88 |  |  |  |  |  |
| **Compassion and empathy** |  |  |  |  |  |  |  |  |  |  |
| Compassion and empathy awareness | 1 | 16 | 69 | 15 | .73 | 0.72 | -2.85 | -1.17 | 1.23 | 2.75 |
| Anticipating and responding to needs | 1 | 47 | 42 | 10 | .69 | 0.77 | -2.63 | -0.05 | 1.47 | 3.24 |
| Dealing with suffering and loss | 2 | 32 | 59 | 8 | .71 | 0.74 | -2.52 | -0.51 | 1.73 | 2.60 |
| *Cronbach’s alpha total subscale* |  |  |  |  | .79 |  |  |  |  |  |
| **Organizational process of care** |  |  |  |  |  |  |  |  |  |  |
| Knowledge of one's own organization | 14 | 42 | 36 | 7 | .76 | 0.71 | -1.37 | 0.19 | 1.94 | 1.76 |
| Knowledge of other organizations involved in care | 9 | 47 | 34 | 10 | .76 | 0.72 | -1.74 | 0.20 | 1.57 | 1.89 |
| Task differentiation/ reallocation | 4 | 35 | 48 | 13 | .77 | 0.68 | -2.33 | -0.38 | 1.48 | 1.71 |
| Collaboration in the organizational process of care | 5 | 46 | 40 | 9 | .75 | 0.77 | -1.98 | 0.03 | 1.59 | 2.51 |
| *Cronbach’s alpha total subscale* |  |  |  |  | .81 |  |  |  |  |  |
| *Cronbach’s alpha for the total job domain 2* |  |  |  |  | .92 |  |  |  |  |  |
| **Job domain 3: Managing & Improving** | | | | | | | | | | |
| **Coordination of care** |  |  |  |  |  |  |  |  |  |  |
| Estimating the necessary care | 1 | 21 | 51 | 27 | .80 | 0.75 | -2.65 | -0.93 | 0.70 | 2.48 |
| Time management and planning | 5 | 38 | 36 | 21 | .81 | 0.70 | -2.26 | -0.17 | 0.99 | 1.60 |
| Continuity of care | 3 | 45 | 41 | 10 | .79 | 0.80 | -2.11 | -0.05 | 1.45 | 3.02 |
| Cost-awareness, avoiding waste | 14 | 43 | 36 | 7 | .83 | 0.64 | -1.51 | 0.22 | 2.16 | 1.33 |
| Care coordination | 1 | 30 | 48 | 20 | .81 | 0.72 | -2.95 | -0.61 | 1.01 | 2.07 |
| *Cronbach’s alpha total subscale* |  |  |  |  | .84 |  |  |  |  |  |
| **Patient safety culture** |  |  |  |  |  |  |  |  |  |  |
| Patient safety and the Patient Safety Management System | 8 | 49 | 37 | 7 | .78 | 0.67 | -2.03 | 0.25 | 2.04 | 1.53 |
| Dealing with conflicts with patients and/or next-of-kin | 6 | 44 | 33 | 17 | .74 | 0.78 | -1.88 | 0.02 | 1.06 | 2.59 |
| Dealing with conflicts with colleagues and other staff | 6 | 57 | 29 | 8 | .76 | 0.73 | -1.87 | 0.45 | 1.64 | 2.38 |
| Preventing mistakes and near misses | 1 | 42 | 43 | 14 | .77 | 0.70 | -3.28 | -0.19 | 1.36 | 1.80 |
| *Cronbach’s alpha total subscale* |  |  |  |  | .81 |  |  |  |  |  |
| **Quality of care** |  |  |  |  |  |  |  |  |  |  |
| Guidelines, professional standards and protocols | 2 | 38 | 47 | 13 | .85 | 0.70 | -2.65 | -0.3 | 1.45 | 1.97 |
| Clinically uncertain situations and EBP (Evidence-Based Practice) nursing | 15 | 39 | 40 | 6 | .85 | 0.72 | -1.25 | 0.07 | 1.97 | 1.91 |
| Quality systems | 13 | 51 | 29 | 8 | .83 | 0.81 | -1.31 | 0.39 | 1.59 | 2.94 |
| Quality of Care | 7 | 49 | 35 | 9 | .83 | 0.79 | -1.69 | 0.2 | 1.58 | 2.69 |
| Measuring quality | 16 | 45 | 28 | 10 | .84 | 0.75 | -1.20 | 0.39 | 1.52 | 2.05 |
| *Cronbach’s alpha total subscale* |  |  |  |  | .87 |  |  |  |  |  |
| **Social action** |  |  |  |  |  |  |  |  |  |  |
| Epidemiology | 11 | 70 | 14 | 5 | .73 | 0.72 | -1.52 | 1.14 | 1.81 | 2.38 |
| Risk prevention and health education | 4 | 60 | 28 | 8 | .70 | 0.78 | -2.03 | 0.44 | 1.58 | 2.82 |
| Identifying ethical and moral dilemmas | 3 | 45 | 45 | 8 | .76 | 0.60 | -3.03 | -0.06 | 2.09 | 1.40 |
| Developments in society relevant to healthcare | 21 | 43 | 29 | 6 | .74 | 0.69 | -1.02 | 0.50 | 2.03 | 1.57 |
| *Cronbach’s alpha total subscale* |  |  |  |  | .79 |  |  |  |  |  |
| **Knowledge and science** |  |  |  |  |  |  |  |  |  |  |
| Evidence-Based Practice (EBP) | 21 | 40 | 36 | 3 | .83 | 0.75 | -0.95 | 0.32 | 2.38 | 2.16 |
| Looking up information on Evidence and Evidence-Based protocols | 20 | 43 | 30 | 6 | .82 | 0.80 | -0.95 | 0.39 | 1.77 | 2.61 |
| Current scientific developments | 9 | 68 | 19 | 4 | .85 | 0.66 | -1.83 | 1.04 | 2.07 | 1.85 |
| Review of nursing research and EBP articles | 31 | 33 | 21 | 16 | .83 | 0.79 | -0.53 | 0.45 | 1.05 | 2.19 |
| Multidisciplinary scientific medical research | 26 | 52 | 19 | 2 | .83 | 0.77 | -0.75 | 0.96 | 2.35 | 2.47 |
| *Cronbach’s alpha total subscale* |  |  |  |  | .86 |  |  |  |  |  |
| **Professionalism** |  |  |  |  |  |  |  |  |  |  |
| Reflecting on your own actions and knowledge | 1 | 34 | 52 | 13 | .78 | 0.70 | -3.26 | -0.49 | 1.44 | 2.03 |
| Increasing expertise | 14 | 37 | 38 | 12 | .80 | 0.64 | -1.51 | -0.02 | 1.68 | 1.22 |
| Guiding and supporting new colleagues and trainees | 10 | 34 | 40 | 16 | .79 | 0.66 | -1.74 | -0.23 | 1.33 | 1.36 |
| Professional code in nursing | 3 | 41 | 48 | 9 | .77 | 0.76 | -2.22 | -0.21 | 1.65 | 2.65 |
| Legislation and regulations applicable to nursing | 10 | 51 | 32 | 7 | .77 | 0.73 | -1.63 | 0.35 | 1.80 | 2.07 |
| *Cronbach’s alpha total subscale* |  |  |  |  | .82 |  |  |  |  |  |
| *Cronbach’s alpha for the total job domain 3* |  |  |  |  | .92 |  |  |  |  |  |
| *Cronbach’s alpha for the total FAN* |  |  |  |  | .94 |  |  |  |  |  |

Note: The meaning of 0.00 is not always 0 but <0.005, due to the use of two decimals.
