# Supplementary material for: Nurses’ Professional Performance: The Development and Evaluation of a Formative Workplace-Based Self-Assessment Instrument
Source: Int J Nurs Stud Adv. 2026 May 14;10:100542. doi: 10.1016/j.ijnsa.2026.100542 (PMC13196436; doi:10.1016/j.ijnsa.2026.100542)
Supplement: Supplementary file 4 [file mmc4.docx]

**Appendix, Table 5. Evaluation of the FAN along the ‘Eight Validity Challenges**

|  |
| --- |

| Table 5. ‘Eight Validity Challenges’ of Mislevy (2016) with response on how nurses’ capabilities are harnessed along these validity challenges in the Formative Assessment for Nurses’ Professional Performance (FAN) instrument during Evidence-Centered Design stages 1 to 4. | |
| --- | --- |
| **Validity Challenge** | **Responses on how nurses’ capabilities are harnassed along these validity challenges.** |
| 1. What do we want to assess? | Performances at a detailed task level, verifiable, observable, attainable, and measurable behavior level (i.e., constituent skills and performances), differentiated by beginner, competent, proficient, and expert levels of (pre)graduated/registered nurses, which comprise the nursing profession in a variety of workplaces for development purposes and optimization of the staffing mix (Merriënboer & Kirschner 2018). |
| 1. What kinds of performances do we need to observe, in what kinds of situations? | Verifiable professional tasks of nurses across a variety of job roles and workplaces, at beginner, competent, proficient, and expert levels, in routine and complex non-routine situations, from recurrent automated situations to non-recurrent situations. |
| 1. How should we think about constructs? | To design performance indicators that harness nurses’ capabilities across four answer categories on a cumulative scale. To discriminate among the answer categories across four performance levels (beginner, competent, proficient, and expert), with detailed constituent task and behavior descriptions from a ‘Whole Task’ perspective, which together are intended to comprise nurses’ work(Merriënboer & Kirschner 2018). Unlike a commonly used Likert-style scale with competence statements described in the items for self-assessment in a Likert-style answer category. |
| 1. How do we assess higher order skills? | To include in the task descriptions action verbs from the Revised Taxonomy of Bloom to measure higher-order thinking skills, with metacognition as the highest level, to benefit others in a professional leadership role and to be able to direct and delegate (Krathwohl, 2002). |
| 1. What is the role of measurement models? | There are two main functions of assessments, ‘Assessment of Learning’ and ‘Assessment for Learning’. The purpose of the Formative Assessment for Nurses is ‘Assessment for Learning’, structured as an educational sequence of ‘Whole Tasks’ descriptions from beginner to expert, and after scoring, it serves as a guide for continuous professional development to attain the sufficient level and/or to keep well equipped. The task description(s) from beginner, competent, proficient to expert level turn at that moment into learning task descriptions (i.e., learning goals) to attain. Subsequently, it serves as a needs assessment, and the necessary teaching and learning can be centered around those selected learning tasks in order to obtain the desired performance level in the real world or the required standard. |
| 1. How do we “score” complex, interactive, performances at scale? | To “score” complexity, the different categories of lower- and higher-order ‘action verbs’ from the Taxonomy of Bloom (Krathwohl, 2002; Anderson & Krathwohl, 2001) are integrated into the constituent ‘Whole Task’ descriptions, reflecting an increase from standard to (more) complex situations, from recurrent automated situations to non-recurrent situations. While filling in the assessment, nurses read the constituent task descriptions in sequence from beginner to expert level, reflect on each level to determine how well it reflects their perceived performance in the workplace, and select the achieved performance level answer category for that moment to score. Then, nurses read beyond their scored performance level to gain awareness of the next performance level and score this level as a goal for development when desired. Interaction means verifying the result with the team leader, educators, and colleagues using examples of casuistry (i.e., validity arguments), deriving evidence, discussing progression in their profession, determining what part of the assessment to include in a professional or team development plan to improve individual or collaborative performance, and organizing the next level ‘Whole Tasks’ for learning and practice in patient care. Nurses engage dynamically with what they do in practice and, together with others, provide sources of evidence. These interactions, along the construct that is assessed, lead to validity arguments and to the legitimacy of a next step in their career. |
| 1. How do we assess interactional skills? | The assessment covers social, inter-, intra-, and multidisciplinary communicative and collaborative interactions, both horizontal and vertical. These include beginners asking for help, experts offering help, colleagues supporting each other, teamwork, deliberate practice, negotiation, and coordination in contexts where the professional’s ‘Whole Task’ involves others. These performances are measured by integrating social and collaborative interactions into the ‘Whole Task’ descriptions, where applicable, across performance levels. The validity argument and evidence are established after the assessment is completed. |
| 1. How do we take advantage of complex performance tasks? | The higher a nurse's score on the cumulative scale, the more complex the professional tasks are, as described by constituent knowledge, skills, and attitudes that measure what they do at what performance level. Mastery at the expert level enables a nurse to perform at a leadership level within and between teams, across disciplines. |
