# Supplementary material for: Nurses’ Professional Performance: The Development and Evaluation of a Formative Workplace-Based Self-Assessment Instrument
Source: Int J Nurs Stud Adv. 2026 May 14;10:100542. doi: 10.1016/j.ijnsa.2026.100542 (PMC13196436; doi:10.1016/j.ijnsa.2026.100542)
Supplement: Supplementary file 5 [file mmc5.docx]

**Appendix, Tables 3a, 3b, 3c.** **ANOVA regarding nurses’ job roles.**

| Table 3a. *Analysis of Variance (ANOVA) test between and within groups of nurses performance levels, measured with the Formative Assessment for Nurses’ Professional Performance, and their job roles with the sum of squares, degrees of freedom, mean square, F-value and P-value over the total* Formative Assessment for Nurses' Professional Performance instrument and post-hoc Tamhane tests | | | | | | |
| --- | --- | --- | --- | --- | --- | --- |
|  | | Sum of Squares | df | Mean Square | F | Sig. |
| Total Formative Assessment for Nurses’ Professional Performance instrument | Between Groups | 121.184 | 5 | 24.237 | 108.930 | <.001 |
|  | Within Groups | 1202.389 | 5404 | .222 |  |  |
|  | Total | 1323.573 | 5409 |  |  |  |

| Table 3b. *Analysis of Variance (ANOVA*) *effect sizes^a^ between and within groups of nurse’ performance levels and their job roles with point estimate and confidence interval over the total* Formative Assessment for Nurses' Professional Performance *instrument.* | | | | |
| --- | --- | --- | --- | --- |
|  | | Point Estimate | 95% Confidence Interval | |
|  |  |  | Lower | Upper |
| Total Formative Assessment for Nurses’ Professional Performance instrument | Eta-squared | .092 | .077 | .106 |
|  | Epsilon-squared | .091 | .076 | .105 |
|  | Omega-squared Fixed-effect | .091 | .076 | .105 |
|  | Omega-squared Random-effect | .020 | .016 | .023 |
| a. Eta-squared and Epsilon-squared are estimated based on the fixed-effect model. | | | | |

| Table 3c. Post-hoc ANOVA pairwise mean differences between job roles with standard errors, Tamhane corrected p-value and confidence intervals. | | | | | | | |
| --- | --- | --- | --- | --- | --- | --- | --- |
| Dependent Variable | (I) Job role | (J) Job role | Mean Difference (I-J) | Std. Error | Sig. | 95% Confidence Interval | |
|  |  |  |  |  |  | Lower Bound | Upper Bound |
| Total Formative Assessment for Nurses’ Professional Performance instrument | General nurse | Specialized nurse | -.25423^*^ | .01420 | .000 | -.2958 | -.2126 |
|  |  | Nurse coordinator | -.39317^*^ | .03363 | .000 | -.4926 | -.2937 |
|  |  | Senior nurse | -.37440^*^ | .02613 | .000 | -.4513 | -.2975 |
|  |  | Nurse consultant | -.35598^*^ | .07374 | <.001 | -.5812 | -.1307 |
|  |  | Nurse practitioner | -.42648^*^ | .07907 | <.001 | -.6767 | -.1763 |
|  | Specialized nurse | General nurse | .25423^*^ | .01420 | .000 | .2126 | .2958 |
|  |  | Nurse coordinator | -.13895^*^ | .03444 | .001 | -.2407 | -.0372 |
|  |  | Senior nurse | -.12017^*^ | .02716 | <.001 | -.2000 | -.0403 |
|  |  | Nurse consultant | -.10175 | .07411 | .944 | -.3279 | .1244 |
|  |  | Nurse practitioner | -.17225 | .07941 | .437 | -.4232 | .0787 |
|  | Nurse coordinator | General nurse | .39317^*^ | .03363 | .000 | .2937 | .4926 |
|  |  | Specialized nurse | .13895 | .03444 | .001 | .0372 | .2407 |
|  |  | Senior nurse | .01878 | .04083 | 1.000 | -.1014 | .1390 |
|  |  | Nurse consultant | .03720 | .08014 | 1.000 | -.2047 | .2791 |
|  |  | Nurse practitioner | -.03330 | .08506 | 1.000 | -.2972 | .2306 |
|  | Senior nurse | General nurse | .37440^*^ | .02613 | .000 | .2975 | .4513 |
|  |  | Specialized nurse | .12017^*^ | .02716 | <.001 | .0403 | .2000 |
|  |  | Nurse coordinator | -.01878 | .04083 | 1.000 | -.1390 | .1014 |
|  |  | Nurse consultant | .01842 | .07729 | 1.000 | -2159 | .2528 |
|  |  | Nurse practitioner | -.05208 | .08239 | 1.000 | -.3097 | .2055 |
|  | Nurse consultant | General nurse | .35598^*^ | .07374 | <.001 | .1307 | .5812 |
|  |  | Specialized nurse | .10175 | .07411 | .944 | -.1244 | .3279 |
|  |  | Nurse coordinator | -.03720 | .08014 | 1.000 | -.2791 | .2047 |
|  |  | Senior nurse | -.01842 | .07729 | 1.000 | -.2528 | .2159 |
|  |  | Nurse practitioner | -.07050 | .10744 | 1.000 | -.3952 | .2542 |
|  | Nurse practitioner | General nurse | .42648^*^ | .07907 | <.001 | .1763 | .6767 |
|  |  | Specialized nurse | .17225 | .07941 | .437 | -.0787 | .4232 |
|  |  | Nurse coordinator | .03330 | .08506 | 1.000 | -.2306 | 2972 |
|  |  | Senior nurse | .05208 | .08239 | 1.000 | -.2055 | .3097 |
|  |  | Nurse consultant | .07050 | .10744 | 1.000 | -2542 | 3952 |
| *. The mean difference is statistically significant at the 0.05 level. | | | | | | | |

|  |
| --- |
