# Supplementary material for: Nurses’ Professional Performance: The Development and Evaluation of a Formative Workplace-Based Self-Assessment Instrument
Source: Int J Nurs Stud Adv. 2026 May 14;10:100542. doi: 10.1016/j.ijnsa.2026.100542 (PMC13196436; doi:10.1016/j.ijnsa.2026.100542)
Supplement: Supplementary file 6 [file mmc6.docx]

**Appendix, Tables 4a, 4b, 4c. ANOVA regarding nurses’ workplaces.**

|  | | | | | | | |
| --- | --- | --- | --- | --- | --- | --- | --- |
| Table 4a. *Analysis of Variance (ANOVA) test between and within groups of nurses performance levels, measured with the Formative Assessment for Nurses’ Professional Performance, and their workplace with the sum of squares, degrees of freedom, mean square, F-value and P-value over the total* Formative Assessment for Nurses' Professional Performance instrument and post-hoc Tamhane tests | | | | | | |  |
|  | | Sum of Squares | df | Mean Square | F | Sig. |  |
| Total Formative Assessment for Nurses’ Professional Performance instrument | Between Groups | 49.680 | 13 | 3.822 | 16.190 | <.001 |  |
|  | Within Groups | 1273.912 | 5397 | .236 |  |  |  |
|  | Total | 1323.592 | 5410 |  |  |  |  |

| Table 4b. *Analysis of Variance (ANOVA*) *effect sizes^a^ between and within groups of nurse’ performance levels and their workplace with point estimate and confidence interval over the total* Formative Assessment for Nurses' Professional Performance *instrument..* | | | | |
| --- | --- | --- | --- | --- |
|  | | Point Estimate | 95% Confidence Interval | |
|  |  |  | Lower | Upper |
| Total Formative Assessment for Nurses’ Professional Performance instrument | Eta-squared | .038 | .026 | .046 |
|  | Epsilon-squared | .035 | .024 | .043 |
|  | Omega-squared Fixed-effect | .035 | .024 | .043 |
|  | Omega-squared Random-effect | .003 | .002 | .003 |
| a. Eta-squared and Epsilon-squared are estimated based on the fixed-effect model. | | | | |

| Table 4c. Post-hoc ANOVA pairwise mean differences between workplace (i.e., type of care classification) with standard errors, Tamhane corrected p-value and confidence intervals. | | | | | | |
| --- | --- | --- | --- | --- | --- | --- |
|  |  |  |  |  |  | |
| (I) Type of care classification | (J) Type of care classification | Mean Difference (I-J) | Std. Error | Sig. | 95% Confidence Interval | |
|  |  |  |  |  | Lower Bound | Upper Bound |
| Acute care - ICU ER CCU | Surgical care | .22279^*^ | .02712 | <.001 | .1289 | .3167 |
|  | Non-surgical medical care | .18780^*^ | .02625 | <.001 | .0969 | .2787 |
|  | Woman-mother-child care | .14807^*^ | .02888 | <.001 | .0481 | .2480 |
|  | Acute admission department care | .19327^*^ | .04663 | .005 | .0297 | .3568 |
|  | Dialysis care | .34448^*^ | .04424 | <.001 | .1903 | .4987 |
|  | Rehabilitation care | .31201^*^ | .05915 | <.001 | .1039 | .5201 |
|  | Psychiatry care | -.05504 | .06470 | 1.000 | -.2860 | .1759 |
|  | Geriatrics care | .15025 | .04877 | .196 | -.0210 | .3215 |
|  | Day treatment care | .28595^*^ | .05067 | <.001 | .1076 | .4643 |
|  | Short stay care | .12109 | .07471 | 1.000 | -.1528 | .3949 |
|  | Out patient department care | .19246^*^ | .04986 | .013 | .0186 | .3663 |
|  | Flex pool | .46685^*^ | .03717 | .000 | .3377 | .5960 |
|  | Other | .36834^*^ | .05680 | <.001 | .1685 | .5682 |
| Surgical care | Acute care - ICU ER CCU | -.22279^*^ | .02712 | <.001 | -.3167 | -.1289 |
|  | Non-surgical medical care | -.03499 | .01786 | .991 | -.0967 | .0267 |
|  | Woman-mother-child care | -.07472^*^ | .02154 | .048 | -.1492 | -.0003 |
|  | Acute admission department care | -.02953 | .04248 | 1.000 | -.1796 | .1206 |
|  | Dialysis care | .12169 | .03984 | .207 | -.0178 | .2612 |
|  | Rehabilitation care | .08921 | .05593 | 1.000 | -.1085 | .2869 |
|  | Psychiatry care | -.27783^*^ | .06178 | .002 | -.4998 | -.0558 |
|  | Geriatrics care | -.07255 | .04482 | 1.000 | -.2310 | .0859 |
|  | Day treatment care | .06316 | .04687 | 1.000 | -.1031 | .2294 |
|  | Short stay care | -.10171 | .07219 | 1.000 | -.3688 | .1653 |
|  | Out patient department care | -.03033 | .04600 | 1.000 | -.1913 | .1306 |
|  | Flex pool | .24405^*^ | .03180 | <.001 | .1331 | .3550 |
|  | Other | .14555 | .05345 | .490 | -.0435 | .3346 |
| Non-surgical medical care | Acute care - ICU ER CCU | -.18780^*^ | .02625 | <.001 | -.2787 | -.0969 |
|  | Surgical care | .03499 | .01786 | .991 | -.0267 | .0967 |
|  | Woman-mother-child care | -.03973 | .02044 | .992 | -.1104 | .0309 |
|  | Acute admission department care | .00546 | .04193 | 1.000 | -.1429 | .1538 |
|  | Dialysis care | .15668^*^ | .03926 | .008 | .0191 | .2942 |
|  | Rehabilitation care | .12420 | .05552 | .917 | -.0722 | .3206 |
|  | Psychiatry care | -.24284^*^ | .06140 | .015 | -.4637 | -.0219 |
|  | Geriatrics care | -.03756 | .04430 | 1.000 | -.1944 | .1193 |
|  | Day treatment care | .09815 | .04637 | .966 | -.0665 | .2628 |
|  | Short stay care | -.06672 | .07187 | 1.000 | -.3329 | .1995 |
|  | Out patient department care | .00466 | .04550 | 1.000 | -.1547 | .1640 |
|  | Flex pool | .27904^*^ | .03106 | <.001 | .1705 | .3876 |
|  | Other | .18054 | .05301 | .078 | -.0071 | .3682 |
| Woman-mother-child care | Acute care - ICU ER CCU | -.14807^*^ | .02888 | <.001 | -.2480 | -.0481 |
|  | Surgical care | .07472^*^ | .02154 | .048 | .0003 | .1492 |
|  | Non-surgical medical care | .03973 | .02044 | .992 | -.0309 | .1104 |
|  | Acute admission department care | .04520 | .04363 | 1.000 | -.1086 | .1990 |
|  | Dialysis | .19642^*^ | .04107 | <.001 | .0529 | .3399 |
|  | Rehabilitation | .16394 | .05681 | .338 | -.0366 | .3645 |
|  | Psychiatry | -.20311 | .06257 | .142 | -.4275 | .0213 |
|  | Geriatrics | .00218 | .04591 | 1.000 | -.1598 | .1641 |
|  | Day treatment | .13788 | .04791 | .349 | -.0316 | .3074 |
|  | Short stay | -.02698 | .07288 | 1.000 | -.2958 | .2419 |
|  | Out patient department care | .04439 | .04707 | 1.000 | -.1201 | .2089 |
|  | Flex pool | .31878^*^ | .03332 | .000 | .2027 | .4348 |
|  | Other | .22027^*^ | .05436 | .008 | .0283 | .4122 |
| Acute admission department care | Acute care - ICU ER CCU | -.19327^*^ | .04663 | .005 | -.3568 | -.0297 |
|  | Surgical care | .02953 | .04248 | 1.000 | -.1206 | .1796 |
|  | Non-surgical medical care | -.00546 | .04193 | 1.000 | -.1538 | .1429 |
|  | Woman-mother-child | -.04520 | .04363 | 1.000 | -.1990 | .1086 |
|  | Dialysis | .15122 | .05502 | .443 | -.0409 | .3434 |
|  | Rehabilitation | .11874 | .06759 | 1.000 | -.1179 | .3554 |
|  | Psychiatry | -.24830 | .07250 | .072 | -.5045 | .0079 |
|  | Geriatrics | -.04302 | .05872 | 1.000 | -.2486 | .1625 |
|  | Day treatment | .09268 | .06030 | 1.000 | -.1186 | .3040 |
|  | Short stay | -.07218 | .08156 | 1.000 | -.3662 | .2218 |
|  | Out patient department care | -.00081 | .05963 | 1.000 | -.2088 | .2072 |
|  | Flex pool | .27358^*^ | .04950 | <.001 | .1003 | .4469 |
|  | Other | .17507 | .06554 | .525 | -.0545 | .4046 |
| Dialysis care | Acute care - ICU ER CCU | -.34448^*^ | .04424 | <.001 | -.4987 | -.1903 |
|  | Surgical care | -.12169 | .03984 | .207 | -.2612 | .0178 |
|  | Non-surgical medical care | -.15668^*^ | .03926 | .008 | -.2942 | -.0191 |
|  | Woman-mother-child care | -.19642^*^ | .04107 | <.001 | -.3399 | -.0529 |
|  | Acute admission department care | -.15122 | .05502 | .443 | -.3434 | .0409 |
|  | Rehabilitation care | -.03248 | .06596 | 1.000 | -.2633 | .1984 |
|  | Psychiatry care | -.39952^*^ | .07098 | <.001 | -.6505 | -.1485 |
|  | Geriatrics care | -.19424 | .05684 | .065 | -.3929 | .0044 |
|  | Day treatment care | -.05853 | .05847 | 1.000 | -.2632 | .1461 |
|  | Short stay care | -.22340 | .08021 | .466 | -.5132 | .0664 |
|  | Out patient department care | -.15202 | .05778 | .556 | -.3531 | .0491 |
|  | Flex pool | .12236 | .04726 | .601 | -.0422 | .2870 |
|  | Other | .02386 | .06387 | 1.000 | -.1996 | .2473 |
| Rehabilitation care | Acute care - ICU ER CCU | -.31201^*^ | .05915 | <.001 | -.5201 | -.1039 |
|  | Surgical care | -.08921 | .05593 | 1.000 | -.2869 | .1085 |
|  | Non-surgical medical care | -.12420 | .05552 | .917 | -.3206 | .0722 |
|  | Woman-mother-child care | -.16394 | .05681 | .338 | -.3645 | .0366 |
|  | Acute admission department care | -.11874 | .06759 | 1.000 | -.3554 | .1179 |
|  | Dialysis care | .03248 | .06596 | 1.000 | -.1984 | .2633 |
|  | Psychiatry care | -.36704^*^ | .08112 | .001 | -.6522 | -.0818 |
|  | Geriatrics care | -.16176 | .06908 | .843 | -.4036 | .0801 |
|  | Day treatment care | -.02606 | .07043 | 1.000 | -.2727 | .2206 |
|  | Short stay care | -.19092 | .08930 | .961 | -.5093 | .1275 |
|  | Out patient department care | -.11955 | .06985 | 1.000 | -.3636 | .1245 |
|  | Flex pool | .15484 | .06144 | .684 | -.0608 | .3705 |
|  | Other | .05633 | .07497 | 1.000 | -.2059 | .3185 |
| Psychiatry care | Acute care - ICU ER CCU | .05504 | .06470 | 1.000 | -.1759 | .2860 |
|  | Surgical care | .27783^*^ | .06178 | .002 | .0558 | .4998 |
|  | Non-surgical medical care | .24284^*^ | .06140 | .015 | .0219 | .4637 |
|  | Woman-mother-child care | .20311 | .06257 | .142 | -.0213 | .4275 |
|  | Acute admission department care | .24830 | .07250 | .072 | -.0079 | .5045 |
|  | Dialysis care | .39952^*^ | .07098 | <.001 | .1485 | .6505 |
|  | Rehabilitation care | .36704^*^ | .08112 | .001 | .0818 | .6522 |
|  | Geriatrics care | .20528 | .07389 | .433 | -.0556 | .4661 |
|  | Day treatment care | .34099^*^ | .07515 | .001 | .0758 | .6062 |
|  | Short stay care | .17612 | .09308 | .997 | -.1556 | .5079 |
|  | Out patient department care | .24750 | .07462 | .098 | -.0154 | .5104 |
|  | Flex pool | .52188^*^ | .06680 | <.001 | .2843 | .7594 |
|  | Other | .42338^*^ | .07942 | <.001 | .1439 | .7028 |
| Geriatrics care | Acute care - ICU ER CCU | -.15025 | .04877 | .196 | -.3215 | .0210 |
|  | Surgical care | .07255 | .04482 | 1.000 | -.0859 | .2310 |
|  | Non-surgical medical care | .03756 | .04430 | 1.000 | -.1193 | .1944 |
|  | Woman-mother-child care | -.00218 | .04591 | 1.000 | -.1641 | .1598 |
|  | Acute admission department care | .04302 | .05872 | 1.000 | -.1625 | .2486 |
|  | Dialysis care | .19424 | .05684 | .065 | -.0044 | .3929 |
|  | Rehabilitation care | .16176 | .06908 | .843 | -.0801 | .4036 |
|  | Psychiatry care | -.20528 | .07389 | .433 | -.4661 | .0556 |
|  | Day treatment care | .13570 | .06197 | .935 | -.0814 | .3528 |
|  | Short stay care | -.02916 | .08280 | 1.000 | -.3270 | .2687 |
|  | Out patient department care | .04221 | .06132 | 1.000 | -.1717 | .2562 |
|  | Flex pool | .31660^*^ | .05152 | <.001 | .1361 | .4971 |
|  | Other | .21809 | .06708 | .114 | -.0168 | .4529 |
| Day treatment care | Acute care - ICU ER CCU | -.28595^*^ | .05067 | <.001 | -.4643 | -.1076 |
|  | Surgical care | -.06316 | .04687 | 1.000 | -.2294 | .1031 |
|  | Non-surgical medical care | -.09815 | .04637 | .966 | -.2628 | .0665 |
|  | Woman-mother-child care | -.13788 | .04791 | .349 | -.3074 | .0316 |
|  | Acute admission department care | -.09268 | .06030 | 1.000 | -.3040 | .1186 |
|  | Dialysis care | .05853 | .05847 | 1.000 | -.1461 | .2632 |
|  | Rehabilitation care | .02606 | .07043 | 1.000 | -.2206 | .2727 |
|  | Psychiatry care | -.34099^*^ | .07515 | .001 | -.6062 | -.0758 |
|  | Geriatrics care | -.13570 | .06197 | .935 | -.3528 | .0814 |
|  | Short stay care | -.16486 | .08393 | .993 | -.4663 | .1366 |
|  | Out patient department care | -.09349 | .06283 | 1.000 | -.3130 | .1260 |
|  | Flex pool | .18090 | .05332 | .075 | -.0063 | .3681 |
|  | Other | .08239 | .06847 | 1.000 | -.1574 | .3222 |
| Short stay care | Acute care - ICU ER CCU | -.12109 | .07471 | 1.000 | -.3949 | .1528 |
|  | Surgical care | .10171 | .07219 | 1.000 | -.1653 | .3688 |
|  | Non-surgical medical care | .06672 | .07187 | 1.000 | -.1995 | .3329 |
|  | Woman-mother-child care | .02698 | .07288 | 1.000 | -.2419 | .2958 |
|  | Acute admission department care | .07218 | .08156 | 1.000 | -.2218 | .3662 |
|  | Dialysis care | .22340 | .08021 | .466 | -.0664 | .5132 |
|  | Rehabilitation care | .19092 | .08930 | .961 | -.1275 | .5093 |
|  | Psychiatry care | -.17612 | .09308 | .997 | -.5079 | .1556 |
|  | Geriatrics care | .02916 | .08280 | 1.000 | -.2687 | .3270 |
|  | Day treatment care | .16486 | .08393 | .993 | -.1366 | .4663 |
|  | Out patient department care | .07137 | .08344 | 1.000 | -.2282 | .3709 |
|  | Flex pool | .34576^*^ | .07654 | .003 | .0667 | .6248 |
|  | Other | .24725 | .08777 | .417 | -.0663 | .5608 |
| Out patient department care | Acute care - ICU ER CCU | -.19246^*^ | .04986 | .013 | -.3663 | -.0186 |
|  | Surgical care | .03033 | .04600 | 1.000 | -.1306 | .1913 |
|  | Non-surgical medical care | -.00466 | .04550 | 1.000 | -.1640 | .1547 |
|  | Woman-mother-child care | -.04439 | .04707 | 1.000 | -.2089 | .1201 |
|  | Acute admission department care | .00081 | .05963 | 1.000 | -.2072 | .2088 |
|  | Dialysis care | .15202 | .05778 | .556 | -.0491 | .3531 |
|  | Rehabilitation care | .11955 | .06985 | 1.000 | -.1245 | .3636 |
|  | Psychiatry care | -.24750 | .07462 | .098 | -.5104 | .0154 |
|  | Geriatrics care | -.04221 | .06132 | 1.000 | -.2562 | .1717 |
|  | Day treatment care | .09349 | .06283 | 1.000 | -.1260 | .3130 |
|  | Short stay care | -.07137 | .08344 | 1.000 | -.3709 | .2282 |
|  | Flex pool | .27439^*^ | .05256 | <.001 | .0913 | .4575 |
|  | Other | .17588 | .06788 | .604 | -.0612 | .4130 |
| Flex pool | Acute care - ICU ER CCU | -.46685^*^ | .03717 | .000 | -.5960 | -.3377 |
|  | Surgical care | -.24405^*^ | .03180 | <.001 | -.3550 | -.1331 |
|  | Non-surgical medical care | -.27904^*^ | .03106 | <.001 | -.3876 | -.1705 |
|  | Woman-mother-child care | -.31878^*^ | .03332 | .000 | -.4348 | -.2027 |
|  | Acute admission department care | -.27358^*^ | .04950 | <.001 | -.4469 | -.1003 |
|  | Dialysis care | -.12236 | .04726 | .601 | -.2870 | .0422 |
|  | Rehabilitation care | -.15484 | .06144 | .684 | -.3705 | .0608 |
|  | Psychiatry care | -.52188^*^ | .06680 | <.001 | -.7594 | -.2843 |
|  | Geriatrics care | -.31660^*^ | .05152 | <.001 | -.4971 | -.1361 |
|  | Day treatment care | -.18090 | .05332 | .075 | -.3681 | .0063 |
|  | Short stay care | -.34576^*^ | .07654 | .003 | -.6248 | -.0667 |
|  | Out patient department care | -.27439^*^ | .05256 | <.001 | -.4575 | -.0913 |
|  | Other | -.09850 | .05918 | 1.000 | -.3063 | .1092 |
| Other | Acute care - ICU ER CCU | -.36834^*^ | .05680 | <.001 | -.5682 | -.1685 |
|  | Surgical care | -.14555 | .05345 | .490 | -.3346 | .0435 |
|  | Non-surgical medical care | -.18054 | .05301 | .078 | -.3682 | .0071 |
|  | Woman-mother-child care | -.22027^*^ | .05436 | .008 | -.4122 | -.0283 |
|  | Acute admission department care | -.17507 | .06554 | .525 | -.4046 | .0545 |
|  | Dialysis care | -.02386 | .06387 | 1.000 | -.2473 | .1996 |
|  | Rehabilitation care | -.05633 | .07497 | 1.000 | -.3185 | .2059 |
|  | Psychiatry care | -.42338^*^ | .07942 | <.001 | -.7028 | -.1439 |
|  | Geriatrics care | -.21809 | .06708 | .114 | -.4529 | .0168 |
|  | Day treatment care | -.08239 | .06847 | 1.000 | -.3222 | .1574 |
|  | Short stay care | -.24725 | .08777 | .417 | -.5608 | .0663 |
|  | Out patient department care | -.17588 | .06788 | .604 | -.4130 | .0612 |
|  | Flex pool | .09850 | .05918 | 1.000 | -.1092 | .3063 |
| *. The mean difference is significant at the 0.05 level. | | | | | | |
